# A proposed mechanism for the interaction between the *Candida albicans* Als3 adhesin and streptococcal cell wall proteins

**DOI:** 10.3389/fmicb.2014.00564

**Published:** 2014-11-04

**Authors:** Lois L. Hoyer, Soon-Hwan Oh, Rhian Jones, Ernesto Cota

**Affiliations:** ^1^Department of Pathobiology, University of Illinois at Urbana-ChampaignUrbana, IL, USA; ^2^Department of Life Sciences, Imperial College LondonLondon, UK

**Keywords:** interkingdom interactions, streptococcal adhesin, SspB, Als3 adhesin, peptide-binding cavity, adhesion tethers, isopeptide bond

## Abstract

*C. albicans* binds various bacteria, including the oral commensal *Streptococcus gordonii*. Published reports documented the role of *C. albicans* Als3 and *S. gordonii* SspB in this interaction, and the importance of the Als N-terminal domain (NT-Als) in *C. albicans* adhesion. Here, we demonstrate that Als1 also binds *S. gordonii*. We also describe use of the NT-Als crystal structure to design mutations that precisely disrupt peptide-binding cavity (PBC) or amyloid-forming region (AFR) function in Als3. *C. albicans* displaying Als3 PBC mutant proteins showed significantly reduced binding to *S. gordonii*; mutation of the AFR did not affect the interaction. These observations present an enigma: the Als PBC binds free C termini of ligands, but the SspB C terminus is covalently linked to peptidoglycan and thus unavailable as a ligand. These observations and the predicted SspB elongated structure suggest that partial proteolysis of streptococcal cell wall proteins is necessary for recognition by Als adhesins.

## Introduction

*C. albicans* binds various bacterial species, participating in polymicrobial interactions in the normally healthy host (Shirtliff et al., 2009). One of these is the oral commensal bacterium *Streptococcus gordonii* (Holmes et al., 1996). Co-aggregation between the fungal and bacterial cells is mediated by binding of the adhesive, cell-wall-anchored *S. gordonii* SspB to *C. albicans* Als3 (Silverman et al., 2010). *S. gordonii* cells attach to and accumulate around wild-type *C. albicans* hyphae, but have considerably decreased interaction with hyphae of a Δ*als3/*Δ*als3* strain. Heterologous production of Als3 in the normally non-adherent yeast *Saccharomyces cerevisiae* promotes *S. gordonii* binding to the fungal cells. Deletion of SspA and SspB from *S. gordonii* reduces *S. cerevisiae*/Als3 binding by more than 50%. Heterologous production of SspB in *Lactococcus lactis* promotes binding between the bacterium and the *S. cerevisiae*/Als3 cells. Although residual binding interactions are observed among the various mutant strains, the experimental evidence demonstrated convincingly that SspB and Als3 play a major role in adhesion of *S. gordonii* to *C. albicans*.

Als3 is one of eight *C. albicans* Als proteins (Als1–Als7, Als9), large cell-surface glycoproteins that primarily function in adhesive interactions (Hoyer et al., 2008). While some Als proteins may have overlapping or redundant activity, an understanding of the functional relationships within the Als family is not developed fully. Cell-biology-based inquiry provided an extensive list of divergent binding partners for Als3 including human fibronectin, laminin, collagen, gp96, EGFR, HER2, N-cadherin, E-cadherin, fibrinogen, casein, equine ferritin, bovine serum albumin [reviewed in Lin et al. (2014)], as well as *S. gordonii* SspB (Silverman et al., 2010). The N-terminal domain of Als proteins (NT-Als; approximately 315 aa in the mature protein) is responsible for much of the inferred protein-protein interactions.

Molecular modeling was used to conclude that NT-Als3 interacts with its binding partners by surface-surface interactions (Sheppard et al., 2004; Phan et al., 2007). However, the sheer number and ever-increasing list of proposed binding partners raise the question of how NT-Als domains can adapt to the surface of so many structurally unrelated ligands to establish biologically relevant interactions. Solution of the NT-Als molecular structure (Salgado et al., 2011; Lin et al., 2014) revealed that NT-Als adhesins contain a wide and flat cavity between two immunoglobulin-like domains that can bury up to six C-terminal amino acids of peptides in an extended conformation. The side chain amine of an invariant Lys at the end of this peptide-binding cavity (PBC) establishes a salt bridge with the C-terminal carboxylic acid of the incoming peptide. Thus, NT-Als adhesins have a novel mechanism to bind the flexible C terminus of proteins. The broad specificity of this mechanism can explain Als protein function in biochemical and cell-based assays (Salgado et al., 2011).

NT-Als structural data guided creation of precise site-directed mutations that disrupted function of the NT-Als3 PBC without altering any other aspect of the protein structure (Lin et al., 2014). Full-length *ALS3* genes encoding these mutations were cloned into the *ALS3* locus, resulting in display of the mutant protein on the *C. albicans* cell surface at physiological quantities and native localization (Lin et al., 2014). *C. albicans* strains with disrupted Als3 PBC function had a phenotype identical to the null Δ*als3/*Δ*als3* strain in assays measuring adhesion to monolayers of human pharyngeal epithelial and umbilical vein endothelial cells, and freshly collected human buccal epithelial cells in suspension.

Mutations were also created in the Als amyloid-forming region (AFR), which has been suggested to be involved in *C. albicans* adhesive processes (Lipke et al., 2012). Destruction of the Als3 amyloidogenic potential had little effect on *C. albicans* adhesion to human cell types (Lin et al., 2014). These assays conclusively showed the essential and principal role of the PBC in Als3 adhesion. This system provides a powerful approach to probe Als3 binding functions in its interactions with other proteins. With these reagents and others, we began our effort to understand the interaction between *C. albicans* and *S. gordonii* in greater detail.

## A role for Als proteins, in addition to Als3, in binding of *S. gordonii* to *C. albicans*

**Figure 1:**
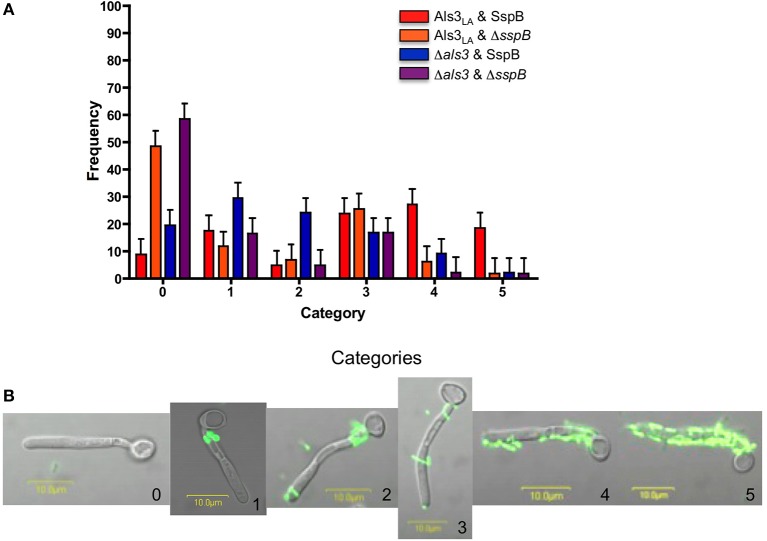
***C. albicans* and *S. gordonii* co-aggregation assays. (A)** Experiments to assess co-aggregation between *C. albicans* and *S. gordonii* were conducted in a shaking flask as described (Silverman et al., 2010), with the exception that *C. albicans* cells from a 16 h yeast extract-peptone-dextrose culture were inoculated into RPMI 1640 medium for 90 min to form hyphae. These growth conditions were chosen because of our detailed knowledge of Als protein localization on cells cultured using this method (Coleman et al., 2009, 2010, 2012; Zhao et al., 2011). *C. albicans* strains included Als3_LA_ (haploid for *ALS3*; encodes one wild-type copy of the *ALS3* large allele from strain SC5314; Lin et al., 2014) and Δ*als3* (strain 1843, Δ*als3/*Δ*als3* null mutant; Zhao et al., 2004). *S. gordonii* strains included SspB (wild type ATCC 10558) and Δ*sspB* (UB1360 Δ(*sspA sspB*); provided by Howard Jenkinson, University of Bristol; Silverman et al., 2010). Following incubation of *C. albicans* with *S. gordonii*, 100 *C. albicans* germ tubes were viewed microscopically and categorized to describe *S. gordonii* binding. The histogram shows the distribution of observations: category 0 (no bacterial cells attached to the germ tube); 1 (bacteria adhered only proximal to the mother yeast, a localization consistent with the involvement of Als1, Als2, and/or Als4); 2 (same as category 1, but with bacteria also at the germ tube tip, an alternate display for Als1); 3 (few bacteria bound diffusely across the germ tube); 4 (approximately half of the germ tube covered in bacteria); 5 (all or nearly all of the germ tube covered in bacteria). For each set of experiments, assays were repeated once or twice, on three or four different occasions. Data were analyzed using a mixed model analysis of variance. The mean for each category within a strain was analyzed using PROC MIXED in SAS (version 9.2, SAS Institute, Inc., Cary, NC). Separation of means was performed with the LSMEANS option. Differences were considered significant at *P* < 0.05. Means and standard errors are reported. The full set of comparisons between means is provided as Table 1 in Supplementary Material. **(B)** Fluorescent micrographs to illustrate the interaction between *C. albicans* and *S. gordonii*. *C. albicans* strain Als3_LA_ was co-incubated with *S. gordonii* strain SspB as described above. Images are labeled with the number corresponding to the category in Figure 1A.

**Figure 2:**
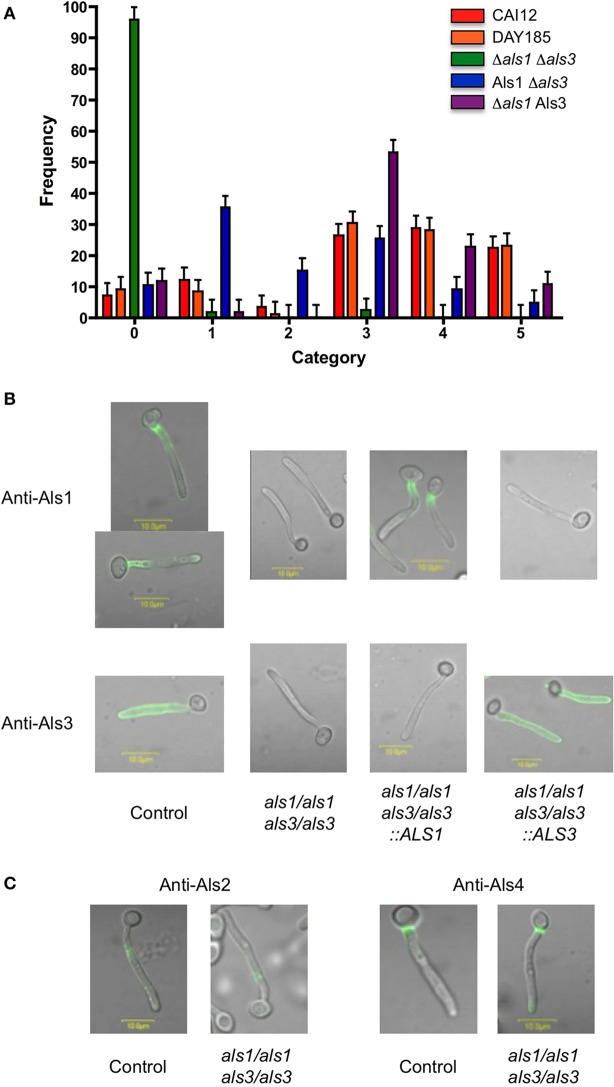
***S. gordonii* co-aggregation with *C. albicans* strains lacking Als1, Als3 or both proteins**. **(A)** The co-aggregation assay was conducted and analyzed as described above. William Fonzi, Georgetown University, provided *C. albicans* control strain CAI12 (*ALS1*/*ALS1 ALS3*/*ALS3*; Porta et al., 1999). Aaron Mitchell, Carnegie Mellon University, provided strains DAY185 (*ALS1*/*ALS1 ALS3*/*ALS3;* Nobile et al., 2008), Δ*als1* Δ*als3* (CJN1348; *als1*/*als1 als3*/*als3*; Nobile et al., 2008), Als1 Δ*als3* (CJN1352; *als1*/*als1*::*ALS1 als3*/*als3*; Nobile et al., 2008), and Δ*als1* Als3 (CJN1356; *als1*/*als1 als3*/*als3*::*ALS3*; Nobile et al., 2008). All assays used *S. gordonii* strain SspB. The full set of comparisons between means is provided as Table 2 in Supplementary Material. **(B)** *C. albicans* hyphae were grown for 90 min, then immunolabeled with a monoclonal antibody that recognizes either Als1 (Coleman et al., 2010) or Als3 (antibody 3-A5; Coleman et al., 2009). Genotypes of each strain are shown below the images. **(C)** *C. albicans* hyphae were grown for 90 min and immunolabeled with a monoclonal antibody that recognizes either Als2 or Als4 (Coleman et al., 2012). Methods for immunolabeling of *C. albicans* germ tubes and for fluorescence microscopy and image processing were published previously (Coleman et al., 2009).

Initial experiments evaluated binding between wild-type and mutant *C. albicans* and *S. gordonii* strains. The interactions were quantified in categories based on the location and abundance of bacteria binding to germ tubes (Figure 1). Co-incubation of control *C. albicans* (Als3_LA_) and *S. gordonii* (SspB) strains showed significantly more *C. albicans* cells with high levels of bacterial adhesion (categories 4 and 5) than when either Als3 or SspB, or both, were absent (compare red bars to all other colors in Figure 1A in categories 4 and 5; *P* < 0.05). Similarly, co-incubation of Als3_LA_ and SspB strains showed fewer category 0 cells than some of the other strain combinations (*P* < 0.0001 compared to Als3_LA_ & Δ*sspB* and Δ*als3* & Δ*sspB*). Interestingly, there was no significant difference in category 0 cells for the combinations of Als3_LA_ & SspB and Δ*als3* & SspB (*P* = 0.2), despite the lack of Als3 in the latter pair. Rather than occupying category 0, cells from the Δ*als3* & SspB combination tended to populate categories 1 and 2, which reflected *S. gordonii* binding to regions of the *C. albicans* germ tube with high abundance of other Als proteins including Als1, Als2, and Als4 (Coleman et al., 2010, 2012; Figure 2). Together, these data confirm the importance of Als3 and SspB in binding of *S. gordonii* to *C. albicans*, and also suggest the involvement of other Als proteins in the interaction.

## *C. albicans* Als1 binds *S. gordonii*

Wild-type *S. gordonii* was incubated with *C. albicans* strains lacking either Als1, Als3, or both proteins (Figure 2A). Als protein localization and abundance on these *C. albicans* strains are shown in Figure 2B. Results for the two control strains (CAI12 and DAY185; wild-type for *ALS1* and *ALS3*) statistically were indistinguishable in each category (*P* > 0.05). Deleting both *ALS3* and *ALS1* (strain Δ*als1* Δ*als3*) resulted in significantly more observations in category 0, and concomitantly, fewer in categories 3, 4 or 5 (*P* < 0.0001 for all comparisons). Only approximately 4% of *C. albicans* cells of the Δ*als1* Δ*als3* strain bound any *S. gordonii*, with observations divided evenly between categories 1 and 3. These results suggested that other Als proteins localized to the *C. albicans* germ tube played an almost undetectable role in the *C. albicans/S. gordonii* interaction. Immunolabeling of the control and double-mutant strains confirmed the presence of Als2 and Als4 in these locations (Figure 2C).

Reintegration of *ALS1* into the Δ*als1* Δ*als3* strain (to produce strain Als1 Δ*als3*) significantly decreased the number of adhesion-negative *C. albicans* cells (Figure 2A, category 0, compare green and blue bars; *P* < 0.0001) and significantly increased the number of *C. albicans* cells in category 1 (*P* < 0.0001) and category 2 (*P* = 0.01). These results supported the conclusion that Als1 functions in adhesion of *S. gordonii* to *C. albicans*. Reintegration of *ALS3* into the Δ*als1* Δ*als3* strain significantly decreased the number of adhesion-negative *C. albicans* cells (Figure 2A, category 0, compare green and purple bars; *P* < 0.0001). The *ALS3* reintegrant strain (Δ*als1* Als3) showed significant increases in cells assigned to categories 3, 4, and 5 compared to the Δ*als1* Δ*als3* strain; these categories reflected locations on the germ tube where Als3 is found (Figure 2B). There were no differences in categories 1 and 2 when comparing the Δ*als1* Δ*als3* strain to the *ALS3* reintegrant (*P* > 0.05). Comparison between results for the *ALS1* and *ALS3* reintegrant strains (Als1 Δ*als3* vs. Δ*als1* Als3) showed no significant difference in category 0, but significant differences between strains in categories that emphasized the effects of protein localization. Overall, these results demonstrated that both Als3 and Als1 participated in adhesion of *S. gordonii* to *C. albicans*, with little contribution from Als2 or Als4, indicating that a common feature of Als proteins, not only present in Als3, is involved in the mechanism of association with SspB.

## The Als3 PBC mediates interaction between *C. albicans* and *S. gordonii* without contribution from the AFR

**Figure 3:**
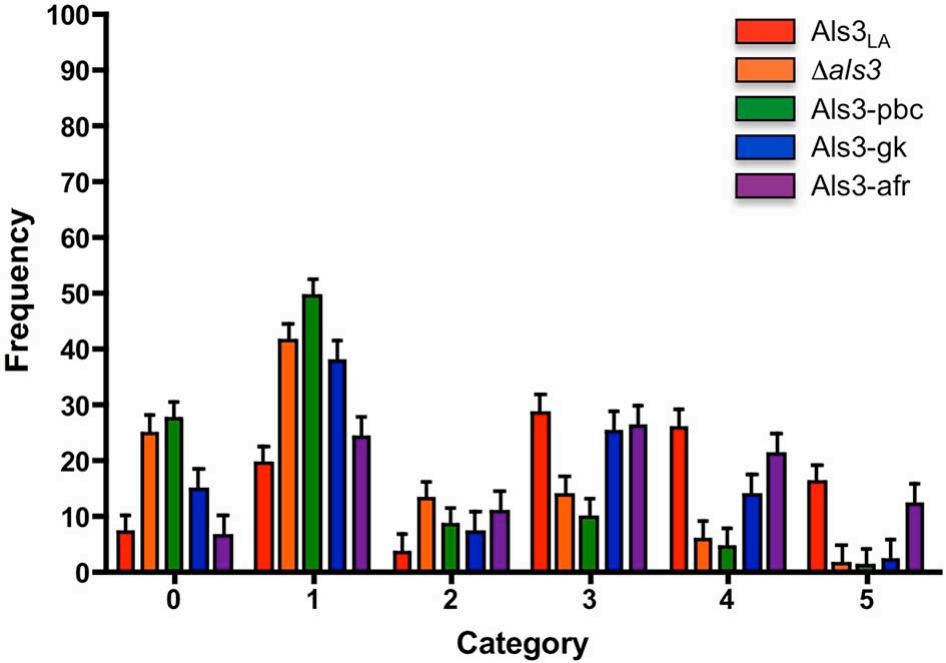
***S. gordonii* co-aggregation with *C. albicans* strains encoding site-directed mutant Als3 alleles**. The co-aggregation assay was conducted and analyzed as described above. *C. albicans* control strains included Als3_LA_ and null Δ*als3*. Experimental strains produced a mutant Als3 protein on the *C. albicans* surface. These included mutations in the PBC (Als3-pbc: K59M, A116V, Y301F; and Als3-gk: S170Y; Lin et al., 2014) and in the AFR (Als3-afr: I311S, I313S; Lin et al., 2014). All assays used *S. gordonii* strain SspB. The full set of comparisons between means is provided as Table 3 in Supplementary Material.

*C. albicans* strains with precise, site-directed Als3 mutations were co-incubated with wild-type *S. gordonii* (SspB) to assess the effect of the PBC and AFR on the cross-kingdom microbial interaction (Figure 3). A control *C. albicans* strain (Als3_LA_) and the Δ*als3* null mutant were included for comparison. Results for the null mutant were similar to those described above: decreased numbers of *C. albicans* cells with abundant *S*. *gordonii* binding (*P* = 0.0001 for category 4, *P* = 0.004 for category 5), an increased frequency of *C. albicans* cells without any bound *S. gordonii* (category 0; *P* = 0.0005) and increased frequency of *C. albicans* with *S. gordonii* bound only in regions rich in Als1 (*P* < 0.0001 for category 1; *P* = 0.05 for category 2).

The Als3-pbc strain displays a mutant Als3 that mimics the “bound” form of the protein (K59M, A116V, Y301F; Lin et al., 2014). Als3-pbc lacks PBC function, while maintaining the surface properties of the wild-type protein. *S. gordonii* binding by strain Als3-pbc was indistinguishable from the null mutant in all categories, emphasizing that Als3-pbc has the same activity as a strain without any Als3. Als3-pbc was significantly different from the control strain in all categories except category 2, a result attributable to the low number of category 2 observations for both isolates. Als3-gk has a “gatekeeper” mutation in Als3 that blocks entry of a peptide ligand to the PBC (Lin et al., 2014). Relative to the control, strain Als3-gk (S170Y; Lin et al., 2014) was enriched for cells in lower category numbers and depleted for cells in higher category numbers, just like Als3-pbc. However, the degree of depletion was not as severe for Als3-gk as for Als3-pbc. Als3 mutations in strain Als3-afr (I311S, I313S; Lin et al., 2014) destroy the amyloidogenic potential of the protein. Als3-afr was indistinguishable from the Als3_LA_ control strain in all categories, suggesting little contribution of the AFR to binding between *C. albicans* and *S. gordonii*.

## Hypothesis and perspectives: how to generate free, flexible SspB C termini for interaction with the PBC of Als proteins?

**Figure 4:**
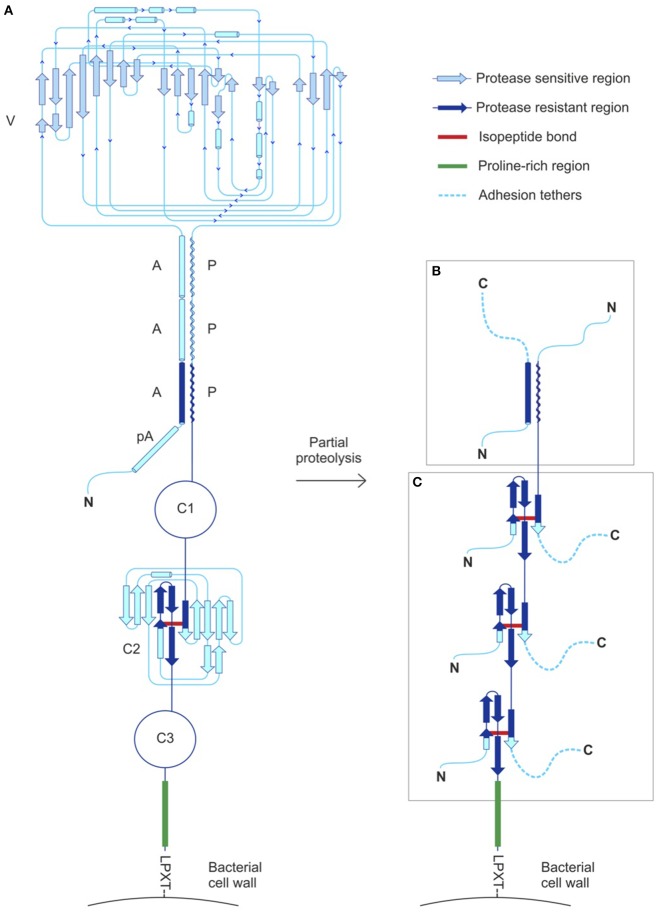
**SspB structural features and proposed mode of interaction with Als proteins. (A)** SspB includes a “pseudo” alpha-helical repeat (pA), three alpha-helical repeats (A), a variable domain (V) and three polyproline-helix repeats (P), followed by three Ig-like domains (C1, C2, and C3), a Pro-rich region and a C-terminal LPxTG motif for sortase-mediated anchoring to the cell wall (Demuth et al., 1990; Forsgren et al., 2010). Structures were solved for two of the three Ig-like domains (C2 and C3), revealing isopeptide bonds formed by residues K1082-N1232 and K1259-N1393, respectively (Forsgren et al., 2010). A topology diagram of C2 is shown as representative of the C domain structure. Structure of the C1 domain was predicted by homology modeling (Forsgren et al., 2010) and shows the potential to form an isopeptide bond between residues K925-N1041. The isopeptide bonds flank large cyclized regions proposed to be substrates for partial proteolysis. **(B)** Adhesion tethers resulting from partial proteolysis of the V domain remain anchored to the streptococcal cell wall by association to the proteolytically resistant AP stalk. **(C)** Partial proteolysis of C domains can also generate adhesion tethers. The proline-rich region immediately N-terminal to the LPxTG motif is likely to be protease-resistant, providing the means to anchor SspB fragments and its numerous potential adhesion tethers to the streptococcal cell wall. Minimally, small remnants of C domains with an isopeptide bond (segments larger than approximately 50 amino acids, shown in dark blue) contain free C termini that provide abundant opportunities for binding *C. albicans* via the Als PBC.

Data presented above demonstrate the primary role of the PBC in mediating interaction between *C. albicans* Als3 and *S. gordonii* SspB. The PBC functions by burying up to six amino acids from flexible C termini of polypeptide ligands (Salgado et al., 2011; Lin et al., 2014). For interactions with host surfaces, it is easy to envision PBC binding to ligands such as extracellular matrix proteins. Less evident, however, is a mechanism for PBC binding to bacterial cell surface proteins such as SspB. The SspB C terminus is not accessible for Als-mediated adhesion, because sortase A cleaves the C-terminal LPxTG recognition sequence and covalently links the exposed Thr to the bacterial peptidoglycan (Nobbs et al., 2007; Figure 4). Furthermore, the sequence of this protein has no detectable “lipobox,” preventing insertion of a diglyceride group for membrane anchoring by the N terminus (Sutcliffe and Harrington, 2002), and the mature form lacks cysteine residues capable to form intermolecular disulfides, as alternative modes of association with the *S. gordonii* cell surface.

Nonetheless, the unusual elongated structure of SspB (157 kDa) provides clues about its potential mode of binding to Als proteins. The SspB globular variable domain (V; Figure 4) is flanked by two non-contiguous A (alanine-rich) and P (polyproline type II) helical regions that associate to form a stalk-like structure (Larson et al., 2010), followed by a C-terminal region containing three tandem Ig-like domains (C1, C2, and C3). Covalent isopeptide bonds between lysine and asparagine residues in the C domains create “cyclized polypeptides” spanning up to 150 amino acids. We hypothesize that selective proteolytic cleavage within the V or C domains generates stable fragments that remain anchored to the cell wall and simultaneously expose multiple C termini, acting as “adhesion tethers” for Als proteins (Figures 4B,C). While *C. albicans* produces an abundance of secreted degradative enzymes that could mediate these activities (Sorgo et al., 2013), proteolytic activity could be of bacterial or even host origin in environments where polymicrobial interactions occur. Although discussions here are focused on SspB, it is important to note that SspB is homologous to SspA and shares the same domain organization with the antigen I/II (AgI/II) family of streptococcal adhesins (Brady et al., 2010). The high level of sequence (Hoyer et al., 2008) and structural (Salgado et al., 2011; Lin et al., 2014) similarity between Als proteins suggests that Als3 functional properties may be extended to the remaining adhesins in the family. Although it remains to be tested directly, it is reasonable to expect that each NT-Als adhesin will have a PBC and display binding sites compatible with a diverse set of C termini created by partial digestion of SspB. More generally, the presence of similar cell-surface proteins in a variety of streptococci provides many potential binding partners, facilitating a broad mechanism for cross-kingdom adhesive interactions.

### Conflict of interest statement

The authors declare that the research was conducted in the absence of any commercial or financial relationships that could be construed as a potential conflict of interest.

## References

[B1] Brady L. J. Maddocks S. E. Larson M. R. Forsgren N. Persson K. Deivanayagam C. C.. (2010). The changing faces of *Streptococcus* antigen I/II polypeptide family adhesins. Mol. Microbiol. 77, 276–286. 10.1111/j.1365-2958.2010.07212.x 20497507 PMC2909373

[B2] Coleman D. A. Oh S.-H. Manfra-Maretta S. L. Hoyer L. L. (2012). A monoclonal antibody specific for *Candida albicans* Als4 demonstrates overlapping localization of Als family proteins on the fungal cell surface and highlights differences between Als localization *in vitro* and *in vivo*. FEMS Immunol. Med. Microbiol. 64, 321–333. 10.1111/j.1574-695X.2011.00914.x 22106872 PMC3299873

[B3] Coleman D. A. Oh S.-H. Zhao X. Hoyer L. L. (2010). Heterogeneous distribution of *Candida albicans* cell-surface antigens demonstrated with an Als1-specific monoclonal antibody. Microbiology 156, 3645–3659. 10.1099/mic.0.043851-0 20705663 PMC3068703

[B4] Coleman D. A. Oh S.-H. Zhao X. Zhao H. Hutchins J. T. Vernachio J. H.. (2009). Monoclonal antibodies specific for *Candida albicans* Als3 that immunolabel fungal cells *in vitro* and *in vivo* and block adhesion to host surfaces. J. Microbiol. Methods 78, 71–78. 10.1016/j.mimet.2009.05.002 19427882 PMC2706517

[B5] Demuth D. R. Golub E. E. Malamud D. (1990). Streptococcal-host interactions. Structural and functional analysis of a *Streptococcus sanguis* receptor for a human salivary glycoprotein. J. Biol. Chem. 265, 7120–7126. 2185241

[B6] Forsgren N. Lamont R. J. Persson K. (2010). Two intramolecular isopeptide bonds are identified in the crystal structure of the *Streptococcus gordonii* SspB C-terminal domain. J. Mol. Biol. 397, 740–751. 10.1016/j.jmb.2010.01.065 20138058 PMC2849992

[B7] Holmes A. R. McNab R. Jenkinson H. F. (1996). *Candida albicans* binding to the oral bacterium *Streptococcus gordonii* involves multiple adhesin-receptor interactions. Infect. Immun. 64, 4680–4685. 8890225 10.1128/iai.64.11.4680-4685.1996 PMC174431

[B8] Hoyer L. L. Green C. B. Oh S.-H. Zhao X. (2008). Discovering the secrets of the *Candida albicans* agglutinin-like sequence (ALS) gene family–a sticky pursuit. Med. Mycol. 46, 1–15. 10.1080/13693780701435317 17852717 PMC2742883

[B9] Larson M. R. Rajashankar K. R. Patel M. H. Robinette R. A. Crowley P. J. Michalek S.. (2010). Elongated fibrillar structure of a streptococcal adhesin assembled by the high-affinity association of alpha- and PPII-helices. Proc. Natl. Acad. Sci. U.S.A. 107, 5983–5988. 10.1073/pnas.0912293107 20231452 PMC2851892

[B10] Lin J. Oh S.-H. Jones R. Garnett J. A. Salgado P. S. Rusnakova S.. (2014). The peptide-binding cavity is essential for Als3-mediated adhesion of *Candida albicans* to human cells. J. Biol. Chem. 289, 18401–18412. 10.1074/jbc.M114.547877 24802757 PMC4140257

[B11] Lipke P. N. Garcia M. C. Alsteens D. Ramsook C. B. Klotz S. A. Dufrene Y. F. (2012). Strengthening relationships: amyloids create adhesion nanodomains in yeasts. Trends Microbiol. 20, 59–65. 10.1016/j.tim.2011.10.002 22099004 PMC3278544

[B12] Nobbs A. H. Vajna R. M. Johnson J. R. Zhang Y. Erlandsen S. L. Oli M. W.. (2007). Consequences of a sortase a mutation in *Streptococcus gordonii*. Microbiology 153, 4088–4097. 10.1099/mic.0.2007/007252-0 18048922

[B13] Nobile C. J. Schneider H. A. Nett J. E. Sheppard D. C. Filler S. G. Andes D. R.. (2008). Complementary adhesin function in *C. albicans* biofilm formation. Curr. Biol. 18, 1017–1024. 10.1016/j.cub.2008.06.034 18635358 PMC2504253

[B14] Phan Q. T. Myers C. L. Fu Y. Sheppard D. C. Yeaman M. R. Welch W. H.. (2007). Als3 is a *Candida albicans* invasin that binds to cadherins and induces endocytosis by host cells. PLoS Biol. 5:e64. 10.1371/journal.pbio.0050064 17311474 PMC1802757

[B15] Porta A. Ramon A. M. Fonzi W. A. (1999). *PRR1*, a homolog of *Aspergillus nidulans* palF, controls pH-dependent gene expression and filamentation in *Candida albicans*. J. Bacteriol. 181, 7516–7523. 10601209 10.1128/jb.181.24.7516-7523.1999 PMC94209

[B16] Salgado P. S. Yan R. Taylor J. D. Burchell L. Jones R. Hoyer L. L.. (2011). Structural basis for the broad specificity to host-cell ligands by the pathogenic fungus *Candida albicans*. Proc. Natl. Acad. Sci. U.S.A. 108, 15775–15779. 10.1073/pnas.1103496108 21896717 PMC3179088

[B17] Sheppard D. C. Yeaman M. R. Welch W. H. Phan Q. T. Fu Y. Ibrahim A. S.. (2004). Functional and structural diversity in the Als protein family of *Candida albicans*. J. Biol. Chem. 279, 30480–30489. 10.1074/jbc.M401929200 15128742

[B18] Shirtliff M. E. Peters B. M. Jabra-Rizk M. A. (2009). Cross-kingdom interactions: *Candida albicans* and bacteria. FEMS Microbiol. Lett. 299, 1–8. 10.1111/j.1574-6968.2009.01668.x 19552706 PMC4406406

[B19] Silverman R. J. Nobbs A. H. Vickerman M. M. Barbour M. E. Jenkinson H. F. (2010). Interaction of *Candida albicans* cell wall Als3 protein with *Streptococcus gordonii* SspB adhesin promotes development of mixed-species communities. Infect. Immun. 78, 4644–4652. 10.1128/IAI.00685-10 20805332 PMC2976310

[B20] Sorgo A. G. Heilmann C. J. Brul S. De Koster C. G. Klis F. M. (2013). Beyond the wall: *Candida albicans* secret(e)s to survive. FEMS Microbiol. Lett. 338, 10–17. 10.1111/1574-6968.12049 23170918

[B21] Sutcliffe I. C. Harrington D. J. (2002). Pattern searches for the identification of putative lipoprotein genes in Gram-positive bacterial genomes. Microbiology 148, 2065–2077. 12101295 10.1099/00221287-148-7-2065

[B22] Zhao X. Oh S.-H. Cheng G. Green C. B. Nuessen J. A. Yeater K.. (2004). *ALS3* and *ALS8* represent a single locus that encodes a *Candida albicans* adhesin; functional comparisons between Als3p and Als1p. Microbiology 150, 2415–2428. 10.1099/mic.0.26943-0 15256583

[B23] Zhao X. Oh S.-H. Coleman D. A. Hoyer L. L. (2011). *ALS51*, a newly discovered gene in the *Candida albicans* ALS family, created by intergenic recombination: analysis of the gene and protein, and implications for evolution of microbial gene families. FEMS Immunol. Med. Microbiol. 61, 245–257. 10.1111/j.1574-695X.2010.00769.x 21208290 PMC3842030

