# Supplementary material for: A proposed mechanism for the interaction between the Candida albicans Als3 adhesin and streptococcal cell wall proteins
Source: Front Microbiol. 2014 Nov 4;5:564. doi: 10.3389/fmicb.2014.00564 (PMC4219490; doi:10.3389/fmicb.2014.00564)
Supplement: Supplementary file 1 [file Table1.PDF]

| **Comparisons Between Means for Fig. 1** | | |
|---|---|---|
| **Observation 1** | **Observation 2** | **P value** |
| Δ*als3* & Δ*sspB*_0 | Δ*als3* & Δ*sspB*_1 | <.0001 |
| Δ*als3* & Δ*sspB*_0 | Δ*als3* & Δ*sspB*_2 | <.0001 |
| Δ*als3* & Δ*sspB*_0 | Δ*als3* & Δ*sspB*_3 | <.0001 |
| Δ*als3* & Δ*sspB*_0 | Δ*als3* & Δ*sspB*_4 | <.0001 |
| Δ*als3* & Δ*sspB*_0 | Δ*als3* & Δ*sspB*_5 | <.0001 |
| Δ*als3* & Δ*sspB*_0 | Δ*als3* & SspB_0 | <.0001 |
| Δ*als3* & Δ*sspB*_0 | Δ*als3* & SspB_1 | 0.0005 |
| Δ*als3* & Δ*sspB*_0 | Δ*als3* & SspB_2 | <.0001 |
| Δ*als3* & Δ*sspB*_0 | Δ*als3* & SspB_3 | <.0001 |
| Δ*als3* & Δ*sspB*_0 | Δ*als3* & SspB_4 | <.0001 |
| Δ*als3* & Δ*sspB*_0 | Δ*als3* & SspB_5 | <.0001 |
| Δ*als3* & Δ*sspB*_0 | Als3$_{LA}$ & Δ*sspB*_0 | 0.2090 |
| Δ*als3* & Δ*sspB*_0 | Als3$_{LA}$ & Δ*sspB*_1 | <.0001 |
| Δ*als3* & Δ*sspB*_0 | Als3$_{LA}$ & Δ*sspB*_2 | <.0001 |
| Δ*als3* & Δ*sspB*_0 | Als3$_{LA}$ & Δ*sspB*_3 | <.0001 |
| Δ*als3* & Δ*sspB*_0 | Als3$_{LA}$ & Δ*sspB*_4 | <.0001 |
| Δ*als3* & Δ*sspB*_0 | Als3$_{LA}$ & Δ*sspB*_5 | <.0001 |
| Δ*als3* & Δ*sspB*_0 | Als3$_{LA}$ & SspB_0 | <.0001 |
| Δ*als3* & Δ*sspB*_0 | Als3$_{LA}$ & SspB_1 | <.0001 |
| Δ*als3* & Δ*sspB*_0 | Als3$_{LA}$ & SspB_2 | <.0001 |
| Δ*als3* & Δ*sspB*_0 | Als3$_{LA}$ & SspB_3 | <.0001 |
| Δ*als3* & Δ*sspB*_0 | Als3$_{LA}$ & SspB_4 | 0.0002 |
| Δ*als3* & Δ*sspB*_0 | Als3$_{LA}$ & SspB_5 | <.0001 |
| Δ*als3* & Δ*sspB*_1 | Δ*als3* & Δ*sspB*_2 | 0.1503 |
| Δ*als3* & Δ*sspB*_1 | Δ*als3* & Δ*sspB*_3 | 0.9762 |
| Δ*als3* & Δ*sspB*_1 | Δ*als3* & Δ*sspB*_4 | 0.0818 |
| Δ*als3* & Δ*sspB*_1 | Δ*als3* & Δ*sspB*_5 | 0.0714 |
| Δ*als3* & Δ*sspB*_1 | Δ*als3* & SspB_0 | 0.7096 |
| Δ*als3* & Δ*sspB*_1 | Δ*als3* & SspB_1 | 0.1054 |
| Δ*als3* & Δ*sspB*_1 | Δ*als3* & SspB_2 | 0.3484 |
| Δ*als3* & Δ*sspB*_1 | Δ*als3* & SspB_3 | 0.9731 |
| Δ*als3* & Δ*sspB*_1 | Δ*als3* & SspB_4 | 0.3582 |
| Δ*als3* & Δ*sspB*_1 | Δ*als3* & SspB_5 | 0.0751 |
| Δ*als3* & Δ*sspB*_1 | Als3$_{LA}$ & Δ*sspB*_0 | 0.0001 |
| Δ*als3* & Δ*sspB*_1 | Als3$_{LA}$ & Δ*sspB*_1 | 0.5594 |
| Δ*als3* & Δ*sspB*_1 | Als3$_{LA}$ & Δ*sspB*_2 | 0.2301 |
| Δ*als3* & Δ*sspB*_1 | Als3$_{LA}$ & Δ*sspB*_3 | 0.2556 |
| Δ*als3* & Δ*sspB*_1 | Als3$_{LA}$ & Δ*sspB*_4 | 0.2037 |
| Δ*als3* & Δ*sspB*_1 | Als3$_{LA}$ & Δ*sspB*_5 | 0.0732 |
| Δ*als3* & Δ*sspB*_1 | Als3$_{LA}$ & SspB_0 | 0.3474 |
| Δ*als3* & Δ*sspB*_1 | Als3$_{LA}$ & SspB_1 | 0.8842 |

| | | |
|---|---|---|
| Δ*als3* & Δ*sspB*_1 | Als3$_{LA}$ & SspB_2 | 0.1453 |
| Δ*als3* & Δ*sspB*_1 | Als3$_{LA}$ & SspB_3 | 0.3572 |
| Δ*als3* & Δ*sspB*_1 | Als3$_{LA}$ & SspB_4 | 0.1835 |
| Δ*als3* & Δ*sspB*_1 | Als3$_{LA}$ & SspB_5 | 0.7935 |
| Δ*als3* & Δ*sspB*_2 | Δ*als3* & Δ*sspB*_3 | 0.1423 |
| Δ*als3* & Δ*sspB*_2 | Δ*als3* & Δ*sspB*_4 | 0.7562 |
| Δ*als3* & Δ*sspB*_2 | Δ*als3* & Δ*sspB*_5 | 0.7076 |
| Δ*als3* & Δ*sspB*_2 | Δ*als3* & SspB_0 | 0.0718 |
| Δ*als3* & Δ*sspB*_2 | Δ*als3* & SspB_1 | 0.0028 |
| Δ*als3* & Δ*sspB*_2 | Δ*als3* & SspB_2 | 0.0192 |
| Δ*als3* & Δ*sspB*_2 | Δ*als3* & SspB_3 | 0.1412 |
| Δ*als3* & Δ*sspB*_2 | Δ*als3* & SspB_4 | 0.5982 |
| Δ*als3* & Δ*sspB*_2 | Δ*als3* & SspB_5 | 0.7256 |
| Δ*als3* & Δ*sspB*_2 | Als3$_{LA}$ & Δ*sspB*_0 | <.0001 |
| Δ*als3* & Δ*sspB*_2 | Als3$_{LA}$ & Δ*sspB*_1 | 0.3884 |
| Δ*als3* & Δ*sspB*_2 | Als3$_{LA}$ & Δ*sspB*_2 | 0.8082 |
| Δ*als3* & Δ*sspB*_2 | Als3$_{LA}$ & Δ*sspB*_3 | 0.0114 |
| Δ*als3* & Δ*sspB*_2 | Als3$_{LA}$ & Δ*sspB*_4 | 0.8645 |
| Δ*als3* & Δ*sspB*_2 | Als3$_{LA}$ & Δ*sspB*_5 | 0.7161 |
| Δ*als3* & Δ*sspB*_2 | Als3$_{LA}$ & SspB_0 | 0.6129 |
| Δ*als3* & Δ*sspB*_2 | Als3$_{LA}$ & SspB_1 | 0.1140 |
| Δ*als3* & Δ*sspB*_2 | Als3$_{LA}$ & SspB_2 | 0.9854 |
| Δ*als3* & Δ*sspB*_2 | Als3$_{LA}$ & SspB_3 | 0.0200 |
| Δ*als3* & Δ*sspB*_2 | Als3$_{LA}$ & SspB_4 | 0.0067 |
| Δ*als3* & Δ*sspB*_2 | Als3$_{LA}$ & SspB_5 | 0.0904 |
| Δ*als3* & Δ*sspB*_3 | Δ*als3* & Δ*sspB*_4 | 0.0769 |
| Δ*als3* & Δ*sspB*_3 | Δ*als3* & Δ*sspB*_5 | 0.0670 |
| Δ*als3* & Δ*sspB*_3 | Δ*als3* & SspB_0 | 0.7319 |
| Δ*als3* & Δ*sspB*_3 | Δ*als3* & SspB_1 | 0.1118 |
| Δ*als3* & Δ*sspB*_3 | Δ*als3* & SspB_2 | 0.3638 |
| Δ*als3* & Δ*sspB*_3 | Δ*als3* & SspB_3 | 0.9969 |
| Δ*als3* & Δ*sspB*_3 | Δ*als3* & SspB_4 | 0.3429 |
| Δ*als3* & Δ*sspB*_3 | Δ*als3* & SspB_5 | 0.0705 |
| Δ*als3* & Δ*sspB*_3 | Als3$_{LA}$ & Δ*sspB*_0 | 0.0002 |
| Δ*als3* & Δ*sspB*_3 | Als3$_{LA}$ & Δ*sspB*_1 | 0.5396 |
| Δ*als3* & Δ*sspB*_3 | Als3$_{LA}$ & Δ*sspB*_2 | 0.2189 |
| Δ*als3* & Δ*sspB*_3 | Als3$_{LA}$ & Δ*sspB*_3 | 0.2681 |
| Δ*als3* & Δ*sspB*_3 | Als3$_{LA}$ & Δ*sspB*_4 | 0.1935 |
| Δ*als3* & Δ*sspB*_3 | Als3$_{LA}$ & Δ*sspB*_5 | 0.0687 |
| Δ*als3* & Δ*sspB*_3 | Als3$_{LA}$ & SspB_0 | 0.3324 |
| Δ*als3* & Δ*sspB*_3 | Als3$_{LA}$ & SspB_1 | 0.9078 |
| Δ*als3* & Δ*sspB*_3 | Als3$_{LA}$ & SspB_2 | 0.1374 |
| Δ*als3* & Δ*sspB*_3 | Als3$_{LA}$ & SspB_3 | 0.3728 |

| | | |
|---|---|---|
| Δ*als3* & Δ*sspB*_3 | $Als3_{LA}$ & SspB_4 | 0.1933 |
| Δ*als3* & Δ*sspB*_3 | $Als3_{LA}$ & SspB_5 | 0.8166 |
| Δ*als3* & Δ*sspB*_4 | Δ*als3* & Δ*sspB*_5 | 0.9484 |
| Δ*als3* & Δ*sspB*_4 | Δ*als3* & SspB_0 | 0.0359 |
| Δ*als3* & Δ*sspB*_4 | Δ*als3* & SspB_1 | 0.0011 |
| Δ*als3* & Δ*sspB*_4 | Δ*als3* & SspB_2 | 0.0085 |
| Δ*als3* & Δ*sspB*_4 | Δ*als3* & SspB_3 | 0.0762 |
| Δ*als3* & Δ*sspB*_4 | Δ*als3* & SspB_4 | 0.4032 |
| Δ*als3* & Δ*sspB*_4 | Δ*als3* & SspB_5 | 0.9676 |
| Δ*als3* & Δ*sspB*_4 | $Als3_{LA}$ & Δ*sspB*_0 | <.0001 |
| Δ*als3* & Δ*sspB*_4 | $Als3_{LA}$ & Δ*sspB*_1 | 0.2422 |
| Δ*als3* & Δ*sspB*_4 | $Als3_{LA}$ & Δ*sspB*_2 | 0.5804 |
| Δ*als3* & Δ*sspB*_4 | $Als3_{LA}$ & Δ*sspB*_3 | 0.0048 |
| Δ*als3* & Δ*sspB*_4 | $Als3_{LA}$ & Δ*sspB*_4 | 0.6307 |
| Δ*als3* & Δ*sspB*_4 | $Als3_{LA}$ & Δ*sspB*_5 | 0.9575 |
| Δ*als3* & Δ*sspB*_4 | $Als3_{LA}$ & SspB_0 | 0.4150 |
| Δ*als3* & Δ*sspB*_4 | $Als3_{LA}$ & SspB_1 | 0.0600 |
| Δ*als3* & Δ*sspB*_4 | $Als3_{LA}$ & SspB_2 | 0.7702 |
| Δ*als3* & Δ*sspB*_4 | $Als3_{LA}$ & SspB_3 | 0.0089 |
| Δ*als3* & Δ*sspB*_4 | $Als3_{LA}$ & SspB_4 | 0.0027 |
| Δ*als3* & Δ*sspB*_4 | $Als3_{LA}$ & SspB_5 | 0.0463 |
| Δ*als3* & Δ*sspB*_5 | Δ*als3* & SspB_0 | 0.0308 |
| Δ*als3* & Δ*sspB*_5 | Δ*als3* & SspB_1 | 0.0009 |
| Δ*als3* & Δ*sspB*_5 | Δ*als3* & SspB_2 | 0.0071 |
| Δ*als3* & Δ*sspB*_5 | Δ*als3* & SspB_3 | 0.0664 |
| Δ*als3* & Δ*sspB*_5 | Δ*als3* & SspB_4 | 0.3681 |
| Δ*als3* & Δ*sspB*_5 | Δ*als3* & SspB_5 | 0.9808 |
| Δ*als3* & Δ*sspB*_5 | $Als3_{LA}$ & Δ*sspB*_0 | <.0001 |
| Δ*als3* & Δ*sspB*_5 | $Als3_{LA}$ & Δ*sspB*_1 | 0.2175 |
| Δ*als3* & Δ*sspB*_5 | $Als3_{LA}$ & Δ*sspB*_2 | 0.5371 |
| Δ*als3* & Δ*sspB*_5 | $Als3_{LA}$ & Δ*sspB*_3 | 0.0040 |
| Δ*als3* & Δ*sspB*_5 | $Als3_{LA}$ & Δ*sspB*_4 | 0.5856 |
| Δ*als3* & Δ*sspB*_5 | $Als3_{LA}$ & Δ*sspB*_5 | 0.9909 |
| Δ*als3* & Δ*sspB*_5 | $Als3_{LA}$ & SspB_0 | 0.3793 |
| Δ*als3* & Δ*sspB*_5 | $Als3_{LA}$ & SspB_1 | 0.0520 |
| Δ*als3* & Δ*sspB*_5 | $Als3_{LA}$ & SspB_2 | 0.7213 |
| Δ*als3* & Δ*sspB*_5 | $Als3_{LA}$ & SspB_3 | 0.0074 |
| Δ*als3* & Δ*sspB*_5 | $Als3_{LA}$ & SspB_4 | 0.0022 |
| Δ*als3* & Δ*sspB*_5 | $Als3_{LA}$ & SspB_5 | 0.0399 |
| Δ*als3* & SspB_0 | Δ*als3* & SspB_1 | 0.2095 |
| Δ*als3* & SspB_0 | Δ*als3* & SspB_2 | 0.5704 |
| Δ*als3* & SspB_0 | Δ*als3* & SspB_3 | 0.7348 |
| Δ*als3* & SspB_0 | Δ*als3* & SspB_4 | 0.1983 |

| | | |
|---|---|---|
| Δ*als3* & SspB_0 | Δ*als3* & SspB_5 | 0.0326 |
| Δ*als3* & SspB_0 | $Als3_{LA}$ & Δ*sspB*_0 | 0.0005 |
| Δ*als3* & SspB_0 | $Als3_{LA}$ & Δ*sspB*_1 | 0.3402 |
| Δ*als3* & SspB_0 | $Als3_{LA}$ & Δ*sspB*_2 | 0.1176 |
| Δ*als3* & SspB_0 | $Als3_{LA}$ & Δ*sspB*_3 | 0.4424 |
| Δ*als3* & SspB_0 | $Als3_{LA}$ & Δ*sspB*_4 | 0.1020 |
| Δ*als3* & SspB_0 | $Als3_{LA}$ & Δ*sspB*_5 | 0.0316 |
| Δ*als3* & SspB_0 | $Als3_{LA}$ & SspB_0 | 0.1912 |
| Δ*als3* & SspB_0 | $Als3_{LA}$ & SspB_1 | 0.8205 |
| Δ*als3* & SspB_0 | $Als3_{LA}$ & SspB_2 | 0.0691 |
| Δ*als3* & SspB_0 | $Als3_{LA}$ & SspB_3 | 0.5821 |
| Δ*als3* & SspB_0 | $Als3_{LA}$ & SspB_4 | 0.3356 |
| Δ*als3* & SspB_0 | $Als3_{LA}$ & SspB_5 | 0.9118 |
| Δ*als3* & SspB_1 | Δ*als3* & SspB_2 | 0.4885 |
| Δ*als3* & SspB_1 | Δ*als3* & SspB_3 | 0.1127 |
| Δ*als3* & SspB_1 | Δ*als3* & SspB_4 | 0.0125 |
| Δ*als3* & SspB_1 | Δ*als3* & SspB_5 | 0.0010 |
| Δ*als3* & SspB_1 | $Als3_{LA}$ & Δ*sspB*_0 | 0.0204 |
| Δ*als3* & SspB_1 | $Als3_{LA}$ & Δ*sspB*_1 | 0.0292 |
| Δ*als3* & SspB_1 | $Als3_{LA}$ & Δ*sspB*_2 | 0.0057 |
| Δ*als3* & SspB_1 | $Als3_{LA}$ & Δ*sspB*_3 | 0.6228 |
| Δ*als3* & SspB_1 | $Als3_{LA}$ & Δ*sspB*_4 | 0.0047 |
| Δ*als3* & SspB_1 | $Als3_{LA}$ & Δ*sspB*_5 | 0.0009 |
| Δ*als3* & SspB_1 | $Als3_{LA}$ & SspB_0 | 0.0118 |
| Δ*als3* & SspB_1 | $Als3_{LA}$ & SspB_1 | 0.1396 |
| Δ*als3* & SspB_1 | $Als3_{LA}$ & SspB_2 | 0.0027 |
| Δ*als3* & SspB_1 | $Als3_{LA}$ & SspB_3 | 0.4778 |
| Δ*als3* & SspB_1 | $Als3_{LA}$ & SspB_4 | 0.7674 |
| Δ*als3* & SspB_1 | $Als3_{LA}$ & SspB_5 | 0.1727 |
| Δ*als3* & SspB_2 | Δ*als3* & SspB_3 | 0.3658 |
| Δ*als3* & SspB_2 | Δ*als3* & SspB_4 | 0.0658 |
| Δ*als3* & SspB_2 | Δ*als3* & SspB_5 | 0.0076 |
| Δ*als3* & SspB_2 | $Als3_{LA}$ & Δ*sspB*_0 | 0.0031 |
| Δ*als3* & SspB_2 | $Als3_{LA}$ & Δ*sspB*_1 | 0.1304 |
| Δ*als3* & SspB_2 | $Als3_{LA}$ & Δ*sspB*_2 | 0.0347 |
| Δ*als3* & SspB_2 | $Als3_{LA}$ & Δ*sspB*_3 | 0.8402 |
| Δ*als3* & SspB_2 | $Als3_{LA}$ & Δ*sspB*_4 | 0.0292 |
| Δ*als3* & SspB_2 | $Als3_{LA}$ & Δ*sspB*_5 | 0.0073 |
| Δ*als3* & SspB_2 | $Als3_{LA}$ & SspB_0 | 0.0629 |
| Δ*als3* & SspB_2 | $Als3_{LA}$ & SspB_1 | 0.4277 |
| Δ*als3* & SspB_2 | $Als3_{LA}$ & SspB_2 | 0.0183 |
| Δ*als3* & SspB_2 | $Als3_{LA}$ & SspB_3 | 0.9863 |
| Δ*als3* & SspB_2 | $Als3_{LA}$ & SspB_4 | 0.6907 |

| | | |
|---|---|---|
| Δ*als3* & SspB_2 | $Als3_{LA}$ & SspB_5 | 0.4979 |
| Δ*als3* & SspB_3 | Δ*als3* & SspB_4 | 0.3410 |
| Δ*als3* & SspB_3 | Δ*als3* & SspB_5 | 0.0700 |
| Δ*als3* & SspB_3 | $Als3_{LA}$ & Δ*sspB*_0 | 0.0002 |
| Δ*als3* & SspB_3 | $Als3_{LA}$ & Δ*sspB*_1 | 0.5371 |
| Δ*als3* & SspB_3 | $Als3_{LA}$ & Δ*sspB*_2 | 0.2175 |
| Δ*als3* & SspB_3 | $Als3_{LA}$ & Δ*sspB*_3 | 0.2697 |
| Δ*als3* & SspB_3 | $Als3_{LA}$ & Δ*sspB*_4 | 0.1922 |
| Δ*als3* & SspB_3 | $Als3_{LA}$ & Δ*sspB*_5 | 0.0681 |
| Δ*als3* & SspB_3 | $Als3_{LA}$ & SspB_0 | 0.3305 |
| Δ*als3* & SspB_3 | $Als3_{LA}$ & SspB_1 | 0.9108 |
| Δ*als3* & SspB_3 | $Als3_{LA}$ & SspB_2 | 0.1364 |
| Δ*als3* & SspB_3 | $Als3_{LA}$ & SspB_3 | 0.3749 |
| Δ*als3* & SspB_3 | $Als3_{LA}$ & SspB_4 | 0.1946 |
| Δ*als3* & SspB_3 | $Als3_{LA}$ & SspB_5 | 0.8196 |
| Δ*als3* & SspB_4 | Δ*als3* & SspB_5 | 0.3809 |
| Δ*als3* & SspB_4 | $Als3_{LA}$ & Δ*sspB*_0 | <.0001 |
| Δ*als3* & SspB_4 | $Als3_{LA}$ & Δ*sspB*_1 | 0.7360 |
| Δ*als3* & SspB_4 | $Als3_{LA}$ & Δ*sspB*_2 | 0.7760 |
| Δ*als3* & SspB_4 | $Als3_{LA}$ & Δ*sspB*_3 | 0.0420 |
| Δ*als3* & SspB_4 | $Als3_{LA}$ & Δ*sspB*_4 | 0.7213 |
| Δ*als3* & SspB_4 | $Als3_{LA}$ & Δ*sspB*_5 | 0.3741 |
| Δ*als3* & SspB_4 | $Als3_{LA}$ & SspB_0 | 0.9832 |
| Δ*als3* & SspB_4 | $Als3_{LA}$ & SspB_1 | 0.2878 |
| Δ*als3* & SspB_4 | $Als3_{LA}$ & SspB_2 | 0.5856 |
| Δ*als3* & SspB_4 | $Als3_{LA}$ & SspB_3 | 0.0683 |
| Δ*als3* & SspB_4 | $Als3_{LA}$ & SspB_4 | 0.0264 |
| Δ*als3* & SspB_4 | $Als3_{LA}$ & SspB_5 | 0.2390 |
| Δ*als3* & SspB_5 | $Als3_{LA}$ & Δ*sspB*_0 | <.0001 |
| Δ*als3* & SspB_5 | $Als3_{LA}$ & Δ*sspB*_1 | 0.2265 |
| Δ*als3* & SspB_5 | $Als3_{LA}$ & Δ*sspB*_2 | 0.5530 |
| Δ*als3* & SspB_5 | $Als3_{LA}$ & Δ*sspB*_3 | 0.0043 |
| Δ*als3* & SspB_5 | $Als3_{LA}$ & Δ*sspB*_4 | 0.6022 |
| Δ*als3* & SspB_5 | $Als3_{LA}$ & Δ*sspB*_5 | 0.9899 |
| Δ*als3* & SspB_5 | $Als3_{LA}$ & SspB_0 | 0.3924 |
| Δ*als3* & SspB_5 | $Als3_{LA}$ & SspB_1 | 0.0549 |
| Δ*als3* & SspB_5 | $Als3_{LA}$ & SspB_2 | 0.7394 |
| Δ*als3* & SspB_5 | $Als3_{LA}$ & SspB_3 | 0.0079 |
| Δ*als3* & SspB_5 | $Als3_{LA}$ & SspB_4 | 0.0024 |
| Δ*als3* & SspB_5 | $Als3_{LA}$ & SspB_5 | 0.0422 |
| $Als3_{LA}$ & Δ*sspB*_0 | $Als3_{LA}$ & Δ*sspB*_1 | <.0001 |
| $Als3_{LA}$ & Δ*sspB*_0 | $Als3_{LA}$ & Δ*sspB*_2 | <.0001 |
| $Als3_{LA}$ & Δ*sspB*_0 | $Als3_{LA}$ & Δ*sspB*_3 | 0.0055 |

| | | |
|---|---|---|
| $Als3_{LA}$ & $\Delta$*sspB*_0 | $Als3_{LA}$ & $\Delta$*sspB*_4 | <.0001 |
| $Als3_{LA}$ & $\Delta$*sspB*_0 | $Als3_{LA}$ & $\Delta$*sspB*_5 | <.0001 |
| $Als3_{LA}$ & $\Delta$*sspB*_0 | $Als3_{LA}$ & SspB_0 | <.0001 |
| $Als3_{LA}$ & $\Delta$*sspB*_0 | $Als3_{LA}$ & SspB_1 | 0.0002 |
| $Als3_{LA}$ & $\Delta$*sspB*_0 | $Als3_{LA}$ & SspB_2 | <.0001 |
| $Als3_{LA}$ & $\Delta$*sspB*_0 | $Als3_{LA}$ & SspB_3 | 0.0029 |
| $Als3_{LA}$ & $\Delta$*sspB*_0 | $Als3_{LA}$ & SspB_4 | 0.0094 |
| $Als3_{LA}$ & $\Delta$*sspB*_0 | $Als3_{LA}$ & SspB_5 | 0.0004 |
| $Als3_{LA}$ & $\Delta$*sspB*_1 | $Als3_{LA}$ & $\Delta$*sspB*_2 | 0.5345 |
| $Als3_{LA}$ & $\Delta$*sspB*_1 | $Als3_{LA}$ & $\Delta$*sspB*_3 | 0.0876 |
| $Als3_{LA}$ & $\Delta$*sspB*_1 | $Als3_{LA}$ & $\Delta$*sspB*_4 | 0.4883 |
| $Als3_{LA}$ & $\Delta$*sspB*_1 | $Als3_{LA}$ & $\Delta$*sspB*_5 | 0.2217 |
| $Als3_{LA}$ & $\Delta$*sspB*_1 | $Als3_{LA}$ & SspB_0 | 0.7202 |
| $Als3_{LA}$ & $\Delta$*sspB*_1 | $Als3_{LA}$ & SspB_1 | 0.4662 |
| $Als3_{LA}$ & $\Delta$*sspB*_1 | $Als3_{LA}$ & SspB_2 | 0.3785 |
| $Als3_{LA}$ & $\Delta$*sspB*_1 | $Als3_{LA}$ & SspB_3 | 0.1347 |
| $Als3_{LA}$ & $\Delta$*sspB*_1 | $Als3_{LA}$ & SspB_4 | 0.0577 |
| $Als3_{LA}$ & $\Delta$*sspB*_1 | $Als3_{LA}$ & SspB_5 | 0.3987 |
| $Als3_{LA}$ & $\Delta$*sspB*_2 | $Als3_{LA}$ & $\Delta$*sspB*_3 | 0.0213 |
| $Als3_{LA}$ & $\Delta$*sspB*_2 | $Als3_{LA}$ & $\Delta$*sspB*_4 | 0.9424 |
| $Als3_{LA}$ & $\Delta$*sspB*_2 | $Als3_{LA}$ & $\Delta$*sspB*_5 | 0.5446 |
| $Als3_{LA}$ & $\Delta$*sspB*_2 | $Als3_{LA}$ & SspB_0 | 0.7921 |
| $Als3_{LA}$ & $\Delta$*sspB*_2 | $Als3_{LA}$ & SspB_1 | 0.1792 |
| $Als3_{LA}$ & $\Delta$*sspB*_2 | $Als3_{LA}$ & SspB_2 | 0.7940 |
| $Als3_{LA}$ & $\Delta$*sspB*_2 | $Als3_{LA}$ & SspB_3 | 0.0361 |
| $Als3_{LA}$ & $\Delta$*sspB*_2 | $Als3_{LA}$ & SspB_4 | 0.0128 |
| $Als3_{LA}$ & $\Delta$*sspB*_2 | $Als3_{LA}$ & SspB_5 | 0.1452 |
| $Als3_{LA}$ & $\Delta$*sspB*_3 | $Als3_{LA}$ & $\Delta$*sspB*_4 | 0.0177 |
| $Als3_{LA}$ & $\Delta$*sspB*_3 | $Als3_{LA}$ & $\Delta$*sspB*_5 | 0.0041 |
| $Als3_{LA}$ & $\Delta$*sspB*_3 | $Als3_{LA}$ & SspB_0 | 0.0401 |
| $Als3_{LA}$ & $\Delta$*sspB*_3 | $Als3_{LA}$ & SspB_1 | 0.3207 |
| $Als3_{LA}$ & $\Delta$*sspB*_3 | $Als3_{LA}$ & SspB_2 | 0.0108 |
| $Als3_{LA}$ & $\Delta$*sspB*_3 | $Als3_{LA}$ & SspB_3 | 0.8269 |
| $Als3_{LA}$ & $\Delta$*sspB*_3 | $Als3_{LA}$ & SspB_4 | 0.8442 |
| $Als3_{LA}$ & $\Delta$*sspB*_3 | $Als3_{LA}$ & SspB_5 | 0.3799 |
| $Als3_{LA}$ & $\Delta$*sspB*_4 | $Als3_{LA}$ & $\Delta$*sspB*_5 | 0.5934 |
| $Als3_{LA}$ & $\Delta$*sspB*_4 | $Als3_{LA}$ & SspB_0 | 0.7371 |
| $Als3_{LA}$ & $\Delta$*sspB*_4 | $Als3_{LA}$ & SspB_1 | 0.1574 |
| $Als3_{LA}$ & $\Delta$*sspB*_4 | $Als3_{LA}$ & SspB_2 | 0.8501 |
| $Als3_{LA}$ & $\Delta$*sspB*_4 | $Als3_{LA}$ & SspB_3 | 0.0304 |
| $Als3_{LA}$ & $\Delta$*sspB*_4 | $Als3_{LA}$ & SspB_4 | 0.0106 |
| $Als3_{LA}$ & $\Delta$*sspB*_4 | $Als3_{LA}$ & SspB_5 | 0.1267 |
| $Als3_{LA}$ & $\Delta$*sspB*_5 | $Als3_{LA}$ & SspB_0 | 0.3854 |

| | | |
|---|---|---|
| $Als3_{LA}$ & Δ*sspB*_5 | $Als3_{LA}$ & SspB_1 | 0.0533 |
| $Als3_{LA}$ & Δ*sspB*_5 | $Als3_{LA}$ & SspB_2 | 0.7299 |
| $Als3_{LA}$ & Δ*sspB*_5 | $Als3_{LA}$ & SspB_3 | 0.0077 |
| $Als3_{LA}$ & Δ*sspB*_5 | $Als3_{LA}$ & SspB_4 | 0.0023 |
| $Als3_{LA}$ & Δ*sspB*_5 | $Als3_{LA}$ & SspB_5 | 0.0410 |
| $Als3_{LA}$ & SspB_0 | $Als3_{LA}$ & SspB_1 | 0.2785 |
| $Als3_{LA}$ & SspB_0 | $Als3_{LA}$ & SspB_2 | 0.6001 |
| $Als3_{LA}$ & SspB_0 | $Als3_{LA}$ & SspB_3 | 0.0653 |
| $Als3_{LA}$ & SspB_0 | $Als3_{LA}$ & SspB_4 | 0.0251 |
| $Als3_{LA}$ & SspB_0 | $Als3_{LA}$ & SspB_5 | 0.2308 |
| $Als3_{LA}$ & SspB_1 | $Als3_{LA}$ & SspB_2 | 0.1100 |
| $Als3_{LA}$ & SspB_1 | $Als3_{LA}$ & SspB_3 | 0.4376 |
| $Als3_{LA}$ & SspB_1 | $Als3_{LA}$ & SspB_4 | 0.2352 |
| $Als3_{LA}$ & SspB_1 | $Als3_{LA}$ & SspB_5 | 0.9076 |
| $Als3_{LA}$ & SspB_2 | $Als3_{LA}$ & SspB_3 | 0.0191 |
| $Als3_{LA}$ & SspB_2 | $Als3_{LA}$ & SspB_4 | 0.0063 |
| $Als3_{LA}$ & SspB_2 | $Als3_{LA}$ & SspB_5 | 0.0871 |
| $Als3_{LA}$ & SspB_3 | $Als3_{LA}$ & SspB_4 | 0.6781 |
| $Als3_{LA}$ & SspB_3 | $Als3_{LA}$ & SspB_5 | 0.5088 |
| $Als3_{LA}$ & SspB_4 | $Als3_{LA}$ & SspB_5 | 0.2835 |
