# Supplementary material for: A proposed mechanism for the interaction between the Candida albicans Als3 adhesin and streptococcal cell wall proteins
Source: Front Microbiol. 2014 Nov 4;5:564. doi: 10.3389/fmicb.2014.00564 (PMC4219490; doi:10.3389/fmicb.2014.00564)
Supplement: Supplementary file 2 [file Table2.PDF]

Comparisons Between Means for Fig. 2		
**Observation 1**	**Observation 2**	**P value**
DAY185_0	DAY185_1	0.9041
DAY185_0	DAY185_2	0.1603
DAY185_0	DAY185_3	0.0003
DAY185_0	DAY185_4	0.0011
DAY185_0	DAY185_5	0.0127
DAY185_0	Δ*als1* Δ*als3*_0	<.0001
DAY185_0	Δ*als1* Δ*als3*_1	0.1983
DAY185_0	Δ*als1* Δ*als3*_2	0.1077
DAY185_0	Δ*als1* Δ*als3*_3	0.2310
DAY185_0	Δ*als1* Δ*als3*_4	0.1077
DAY185_0	Δ*als1* Δ*als3*_5	0.1077
DAY185_0	Als1 Δ*als3*_0	0.7863
DAY185_0	Als1 Δ*als3*_1	<.0001
DAY185_0	Als1 Δ*als3*_2	0.2805
DAY185_0	Als1 Δ*als3*_3	0.0040
DAY185_0	Als1 Δ*als3*_4	1.0000
DAY185_0	Als1 Δ*als3*_5	0.4346
DAY185_0	Δ*als1* Als3_0	0.6301
DAY185_0	Δ*als1* Als3_1	0.1883
DAY185_0	Δ*als1* Als3_2	0.1077
DAY185_0	Δ*als1* Als3_3	<.0001
DAY185_0	Δ*als1* Als3_4	0.0160
DAY185_0	Δ*als1* Als3_5	0.7404
DAY185_0	CAI12_0	0.7404
DAY185_0	CAI12_1	0.5676
DAY185_0	CAI12_2	0.3078
DAY185_0	CAI12_3	0.0026
DAY185_0	CAI12_4	0.0007
DAY185_0	CAI12_5	0.0186
DAY185_1	DAY185_2	0.1983
DAY185_1	DAY185_3	0.0002
DAY185_1	DAY185_4	0.0007
DAY185_1	DAY185_5	0.0092
DAY185_1	Δ*als1* Δ*als3*_0	<.0001
DAY185_1	Δ*als1* Δ*als3*_1	0.2428
DAY185_1	Δ*als1* Δ*als3*_2	0.1357
DAY185_1	Δ*als1* Δ*als3*_3	0.2805
DAY185_1	Δ*als1* Δ*als3*_4	0.1357
DAY185_1	Δ*als1* Δ*als3*_5	0.1357
DAY185_1	Als1 Δ*als3*_0	0.6955
DAY185_1	Als1 Δ*als3*_1	<.0001

DAY185_1	Als1 Δ*als3*_2	0.2310
DAY185_1	Als1 Δ*als3*_3	0.0028
DAY185_1	Als1 Δ*als3*_4	0.9041
DAY185_1	Als1 Δ*als3*_5	0.5082
DAY185_1	Δ*als1* Als3_0	0.5474
DAY185_1	Δ*als1* Als3_1	0.2310
DAY185_1	Δ*als1* Als3_2	0.1357
DAY185_1	Δ*als1* Als3_3	<.0001
DAY185_1	Δ*als1* Als3_4	0.0117
DAY185_1	Δ*als1* Als3_5	0.6516
DAY185_1	CAI12_0	0.8330
DAY185_1	CAI12_1	0.4892
DAY185_1	CAI12_2	0.3677
DAY185_1	CAI12_3	0.0018
DAY185_1	CAI12_4	0.0005
DAY185_1	CAI12_5	0.0137
DAY185_2	DAY185_3	<.0001
DAY185_2	DAY185_4	<.0001
DAY185_2	DAY185_5	0.0002
DAY185_2	Δ*als1* Δ*als3*_0	<.0001
DAY185_2	Δ*als1* Δ*als3*_1	0.9041
DAY185_2	Δ*als1* Δ*als3*_2	0.8330
DAY185_2	Δ*als1* Δ*als3*_3	0.8330
DAY185_2	Δ*als1* Δ*als3*_4	0.8330
DAY185_2	Δ*als1* Δ*als3*_5	0.8330
DAY185_2	Als1 Δ*als3*_0	0.0955
DAY185_2	Als1 Δ*als3*_1	<.0001
DAY185_2	Als1 Δ*als3*_2	0.0148
DAY185_2	Als1 Δ*als3*_3	<.0001
DAY185_2	Als1 Δ*als3*_4	0.1603
DAY185_2	Als1 Δ*als3*_5	0.5276
DAY185_2	Δ*als1* Als3_0	0.0616
DAY185_2	Δ*als1* Als3_1	0.9280
DAY185_2	Δ*als1* Als3_2	0.8330
DAY185_2	Δ*als1* Als3_3	<.0001
DAY185_2	Δ*als1* Als3_4	0.0002
DAY185_2	Δ*als1* Als3_5	0.0845
DAY185_2	CAI12_0	0.2805
DAY185_2	CAI12_1	0.0505
DAY185_2	CAI12_2	0.6955
DAY185_2	CAI12_3	<.0001
DAY185_2	CAI12_4	<.0001
DAY185_2	CAI12_5	0.0003

DAY185_3	DAY185_4	0.6734
DAY185_3	DAY185_5	0.1983
DAY185_3	Δ*als1* Δ*als3*_0	<.0001
DAY185_3	Δ*als1* Δ*als3*_1	<.0001
DAY185_3	Δ*als1* Δ*als3*_2	<.0001
DAY185_3	Δ*als1* Δ*als3*_3	<.0001
DAY185_3	Δ*als1* Δ*als3*_4	<.0001
DAY185_3	Δ*als1* Δ*als3*_5	<.0001
DAY185_3	Als1 Δ*als3*_0	0.0007
DAY185_3	Als1 Δ*als3*_1	0.3677
DAY185_3	Als1 Δ*als3*_2	0.0072
DAY185_3	Als1 Δ*als3*_3	0.3838
DAY185_3	Als1 Δ*als3*_4	0.0003
DAY185_3	Als1 Δ*als3*_5	<.0001
DAY185_3	Δ*als1* Als3_0	0.0013
DAY185_3	Δ*als1* Als3_1	<.0001
DAY185_3	Δ*als1* Als3_2	<.0001
DAY185_3	Δ*als1* Als3_3	0.0001
DAY185_3	Δ*als1* Als3_4	0.1693
DAY185_3	Δ*als1* Als3_5	0.0008
DAY185_3	CAI12_0	<.0001
DAY185_3	CAI12_1	0.0017
DAY185_3	CAI12_2	<.0001
DAY185_3	CAI12_3	0.4706
DAY185_3	CAI12_4	0.7633
DAY185_3	CAI12_5	0.1518
DAY185_4	DAY185_5	0.3838
DAY185_4	Δ*als1* Δ*als3*_0	<.0001
DAY185_4	Δ*als1* Δ*als3*_1	<.0001
DAY185_4	Δ*als1* Δ*als3*_2	<.0001
DAY185_4	Δ*als1* Δ*als3*_3	<.0001
DAY185_4	Δ*als1* Δ*als3*_4	<.0001
DAY185_4	Δ*als1* Δ*als3*_5	<.0001
DAY185_4	Als1 Δ*als3*_0	0.0024
DAY185_4	Als1 Δ*als3*_1	0.1883
DAY185_4	Als1 Δ*als3*_2	0.0216
DAY185_4	Als1 Δ*als3*_3	0.6516
DAY185_4	Als1 Δ*als3*_4	0.0011
DAY185_4	Als1 Δ*als3*_5	<.0001
DAY185_4	Δ*als1* Als3_0	0.0044
DAY185_4	Δ*als1* Als3_1	<.0001
DAY185_4	Δ*als1* Als3_2	<.0001
DAY185_4	Δ*als1* Als3_3	<.0001

DAY185_4	Δ*als1* Als3_4	0.3369
DAY185_4	Δ*als1* Als3_5	0.0028
DAY185_4	CAI12_0	0.0004
DAY185_4	CAI12_1	0.0056
DAY185_4	CAI12_2	<.0001
DAY185_4	CAI12_3	0.7633
DAY185_4	CAI12_4	0.9041
DAY185_4	CAI12_5	0.3078
DAY185_5	Δ*als1* Δ*als3*_0	<.0001
DAY185_5	Δ*als1* Δ*als3*_1	0.0003
DAY185_5	Δ*als1* Δ*als3*_2	<.0001
DAY185_5	Δ*als1* Δ*als3*_3	0.0004
DAY185_5	Δ*als1* Δ*als3*_4	<.0001
DAY185_5	Δ*als1* Δ*als3*_5	<.0001
DAY185_5	Als1 Δ*als3*_0	0.0251
DAY185_5	Als1 Δ*als3*_1	0.0311
DAY185_5	Als1 Δ*als3*_2	0.1436
DAY185_5	Als1 Δ*als3*_3	0.6734
DAY185_5	Als1 Δ*als3*_4	0.0127
DAY185_5	Als1 Δ*als3*_5	0.0014
DAY185_5	Δ*als1* Als3_0	0.0412
DAY185_5	Δ*als1* Als3_1	0.0002
DAY185_5	Δ*als1* Als3_2	<.0001
DAY185_5	Δ*als1* Als3_3	<.0001
DAY185_5	Δ*als1* Als3_4	0.9280
DAY185_5	Δ*als1* Als3_5	0.0290
DAY185_5	CAI12_0	0.0052
DAY185_5	CAI12_1	0.0505
DAY185_5	CAI12_2	0.0007
DAY185_5	CAI12_3	0.5676
DAY185_5	CAI12_4	0.3222
DAY185_5	CAI12_5	0.8803
Δ*als1* Δ*als3*_0	Δ*als1* Δ*als3*_1	<.0001
Δ*als1* Δ*als3*_0	Δ*als1* Δ*als3*_2	<.0001
Δ*als1* Δ*als3*_0	Δ*als1* Δ*als3*_3	<.0001
Δ*als1* Δ*als3*_0	Δ*als1* Δ*als3*_4	<.0001
Δ*als1* Δ*als3*_0	Δ*als1* Δ*als3*_5	<.0001
Δ*als1* Δ*als3*_0	Als1 Δ*als3*_0	<.0001
Δ*als1* Δ*als3*_0	Als1 Δ*als3*_1	<.0001
Δ*als1* Δ*als3*_0	Als1 Δ*als3*_2	<.0001
Δ*als1* Δ*als3*_0	Als1 Δ*als3*_3	<.0001
Δ*als1* Δ*als3*_0	Als1 Δ*als3*_4	<.0001
Δ*als1* Δ*als3*_0	Als1 Δ*als3*_5	<.0001

Δ*als1* Δ*als3*_0	Δ*als1* Als3_0	<.0001
Δ*als1* Δ*als3*_0	Δ*als1* Als3_1	<.0001
Δ*als1* Δ*als3*_0	Δ*als1* Als3_2	<.0001
Δ*als1* Δ*als3*_0	Δ*als1* Als3_3	<.0001
Δ*als1* Δ*als3*_0	Δ*als1* Als3_4	<.0001
Δ*als1* Δ*als3*_0	Δ*als1* Als3_5	<.0001
Δ*als1* Δ*als3*_0	CAI12_0	<.0001
Δ*als1* Δ*als3*_0	CAI12_1	<.0001
Δ*als1* Δ*als3*_0	CAI12_2	<.0001
Δ*als1* Δ*als3*_0	CAI12_3	<.0001
Δ*als1* Δ*als3*_0	CAI12_4	<.0001
Δ*als1* Δ*als3*_0	CAI12_5	<.0001
Δ*als1* Δ*als3*_1	Δ*als1* Δ*als3*_2	0.7404
Δ*als1* Δ*als3*_1	Δ*als1* Δ*als3*_3	0.9280
Δ*als1* Δ*als3*_1	Δ*als1* Δ*als3*_4	0.7404
Δ*als1* Δ*als3*_1	Δ*als1* Δ*als3*_5	0.7404
Δ*als1* Δ*als3*_1	Als1 Δ*als3*_0	0.1210
Δ*als1* Δ*als3*_1	Als1 Δ*als3*_1	<.0001
Δ*als1* Δ*als3*_1	Als1 Δ*als3*_2	0.0201
Δ*als1* Δ*als3*_1	Als1 Δ*als3*_3	<.0001
Δ*als1* Δ*als3*_1	Als1 Δ*als3*_4	0.1983
Δ*als1* Δ*als3*_1	Als1 Δ*als3*_5	0.6089
Δ*als1* Δ*als3*_1	Δ*als1* Als3_0	0.0794
Δ*als1* Δ*als3*_1	Δ*als1* Als3_1	0.9760
Δ*als1* Δ*als3*_1	Δ*als1* Als3_2	0.7404
Δ*als1* Δ*als3*_1	Δ*als1* Als3_3	<.0001
Δ*als1* Δ*als3*_1	Δ*als1* Als3_4	0.0004
Δ*als1* Δ*als3*_1	Δ*als1* Als3_5	0.1077
Δ*als1* Δ*als3*_1	CAI12_0	0.3369
Δ*als1* Δ*als3*_1	CAI12_1	0.0657
Δ*als1* Δ*als3*_1	CAI12_2	0.7863
Δ*als1* Δ*als3*_1	CAI12_3	<.0001
Δ*als1* Δ*als3*_1	CAI12_4	<.0001
Δ*als1* Δ*als3*_1	CAI12_5	0.0004
Δ*als1* Δ*als3*_2	Δ*als1* Δ*als3*_3	0.6734
Δ*als1* Δ*als3*_2	Δ*als1* Δ*als3*_4	1.0000
Δ*als1* Δ*als3*_2	Δ*als1* Δ*als3*_5	1.0000
Δ*als1* Δ*als3*_2	Als1 Δ*als3*_0	0.0616
Δ*als1* Δ*als3*_2	Als1 Δ*als3*_1	<.0001
Δ*als1* Δ*als3*_2	Als1 Δ*als3*_2	0.0085
Δ*als1* Δ*als3*_2	Als1 Δ*als3*_3	<.0001
Δ*als1* Δ*als3*_2	Als1 Δ*als3*_4	0.1077
Δ*als1* Δ*als3*_2	Als1 Δ*als3*_5	0.4003

Δ*als1* Δ*als3*_2	Δ*als1* Als3_0	0.0385
Δ*als1* Δ*als3*_2	Δ*als1* Als3_1	0.7633
Δ*als1* Δ*als3*_2	Δ*als1* Als3_2	1.0000
Δ*als1* Δ*als3*_2	Δ*als1* Als3_3	<.0001
Δ*als1* Δ*als3*_2	Δ*als1* Als3_4	0.0001
Δ*als1* Δ*als3*_2	Δ*als1* Als3_5	0.0540
Δ*als1* Δ*als3*_2	CAI12_0	0.1983
Δ*als1* Δ*als3*_2	CAI12_1	0.0311
Δ*als1* Δ*als3*_2	CAI12_2	0.5474
Δ*als1* Δ*als3*_2	CAI12_3	<.0001
Δ*als1* Δ*als3*_2	CAI12_4	<.0001
Δ*als1* Δ*als3*_2	CAI12_5	0.0002
Δ*als1* Δ*als3*_3	Δ*als1* Δ*als3*_4	0.6734
Δ*als1* Δ*als3*_3	Δ*als1* Δ*als3*_5	0.6734
Δ*als1* Δ*als3*_3	Als1 Δ*als3*_0	0.1436
Δ*als1* Δ*als3*_3	Als1 Δ*als3*_1	<.0001
Δ*als1* Δ*als3*_3	Als1 Δ*als3*_2	0.0251
Δ*als1* Δ*als3*_3	Als1 Δ*als3*_3	<.0001
Δ*als1* Δ*als3*_3	Als1 Δ*als3*_4	0.2310
Δ*als1* Δ*als3*_3	Als1 Δ*als3*_5	0.6734
Δ*als1* Δ*als3*_3	Δ*als1* Als3_0	0.0955
Δ*als1* Δ*als3*_3	Δ*als1* Als3_1	0.9041
Δ*als1* Δ*als3*_3	Δ*als1* Als3_2	0.6734
Δ*als1* Δ*als3*_3	Δ*als1* Als3_3	<.0001
Δ*als1* Δ*als3*_3	Δ*als1* Als3_4	0.0005
Δ*als1* Δ*als3*_3	Δ*als1* Als3_5	0.1282
Δ*als1* Δ*als3*_3	CAI12_0	0.3838
Δ*als1* Δ*als3*_3	CAI12_1	0.0794
Δ*als1* Δ*als3*_3	CAI12_2	0.8566
Δ*als1* Δ*als3*_3	CAI12_3	<.0001
Δ*als1* Δ*als3*_3	CAI12_4	<.0001
Δ*als1* Δ*als3*_3	CAI12_5	0.0006
Δ*als1* Δ*als*3_4	Δ*als1* Δ*als3*_5	1.0000
Δ*als1* Δ*als*3_4	Als1 Δ*als3*_0	0.0616
Δ*als1* Δ*als*3_4	Als1 Δ*als3*_1	<.0001
Δ*als1* Δ*als*3_4	Als1 Δ*als3*_2	0.0085
Δ*als1* Δ*als*3_4	Als1 Δ*als3*_3	<.0001
Δ*als1* Δ*als*3_4	Als1 Δ*als3*_4	0.1077
Δ*als1* Δ*als*3_4	Als1 Δ*als3*_5	0.4003
Δ*als1* Δ*als*3_4	Δ*als1* Als3_0	0.0385
Δ*als1* Δ*als*3_4	Δ*als1* Als3_1	0.7633
Δ*als1* Δ*als*3_4	Δ*als1* Als3_2	1.0000
Δ*als1* Δ*als*3_4	Δ*als1* Als3_3	<.0001

Δ*als1* Δ*als*3_4	Δ*als1* Als3_4	0.0001
Δ*als1* Δ*als*3_4	Δ*als1* Als3_5	0.0540
Δ*als1* Δ*als*3_4	CAI12_0	0.1983
Δ*als1* Δ*als*3_4	CAI12_1	0.0311
Δ*als1* Δ*als*3_4	CAI12_2	0.5474
Δ*als1* Δ*als*3_4	CAI12_3	<.0001
Δ*als1* Δ*als*3_4	CAI12_4	<.0001
Δ*als1* Δ*als*3_4	CAI12_5	0.0002
Δ*als1* Δ*als3*_5	Als1 Δ*als3*_0	0.0616
Δ*als1* Δ*als3*_5	Als1 Δ*als3*_1	<.0001
Δ*als1* Δ*als3*_5	Als1 Δ*als3*_2	0.0085
Δ*als1* Δ*als3*_5	Als1 Δ*als3*_3	<.0001
Δ*als1* Δ*als3*_5	Als1 Δ*als3*_4	0.1077
Δ*als1* Δ*als3*_5	Als1 Δ*als3*_5	0.4003
Δ*als1* Δ*als3*_5	Δ*als1* Als3_0	0.0385
Δ*als1* Δ*als3*_5	Δ*als1* Als3_1	0.7633
Δ*als1* Δ*als3*_5	Δ*als1* Als3_2	1.0000
Δ*als1* Δ*als3*_5	Δ*als1* Als3_3	<.0001
Δ*als1* Δ*als3*_5	Δ*als1* Als3_4	0.0001
Δ*als1* Δ*als3*_5	Δ*als1* Als3_5	0.0540
Δ*als1* Δ*als3*_5	CAI12_0	0.1983
Δ*als1* Δ*als3*_5	CAI12_1	0.0311
Δ*als1* Δ*als3*_5	CAI12_2	0.5474
Δ*als1* Δ*als3*_5	CAI12_3	<.0001
Δ*als1* Δ*als3*_5	CAI12_4	<.0001
Δ*als1* Δ*als3*_5	CAI12_5	0.0002
Als1 Δ*als3*_0	Als1 Δ*als3*_1	<.0001
Als1 Δ*als3*_0	Als1 Δ*als3*_2	0.4173
Als1 Δ*als3*_0	Als1 Δ*als3*_3	0.0085
Als1 Δ*als3*_0	Als1 Δ*als3*_4	0.7863
Als1 Δ*als3*_0	Als1 Δ*als3*_5	0.2940
Als1 Δ*als3*_0	Δ*als1* Als3_0	0.8330
Als1 Δ*als3*_0	Δ*als1* Als3_1	0.1142
Als1 Δ*als3*_0	Δ*als1* Als3_2	0.0616
Als1 Δ*als3*_0	Δ*als1* Als3_3	<.0001
Als1 Δ*als3*_0	Δ*als1* Als3_4	0.0311
Als1 Δ*als3*_0	Δ*als1* Als3_5	0.9520
Als1 Δ*als3*_0	CAI12_0	0.5474
Als1 Δ*als3*_0	CAI12_1	0.7633
Als1 Δ*als3*_0	CAI12_2	0.1983
Als1 Δ*als3*_0	CAI12_3	0.0056
Als1 Δ*als3*_0	CAI12_4	0.0017
Als1 Δ*als3*_0	CAI12_5	0.0359

Als1 Δ*als3*_1	Als1 Δ*als3*_2	0.0005
Als1 Δ*als3*_1	Als1 Δ*als3*_3	0.0794
Als1 Δ*als3*_1	Als1 Δ*als3*_4	<.0001
Als1 Δ*als3*_1	Als1 Δ*als3*_5	<.0001
Als1 Δ*als3*_1	Δ*als1* Als3_0	<.0001
Als1 Δ*als3*_1	Δ*als1* Als3_1	<.0001
Als1 Δ*als3*_1	Δ*als1* Als3_2	<.0001
Als1 Δ*als3*_1	Δ*als1* Als3_3	0.0020
Als1 Δ*als3*_1	Δ*als1* Als3_4	0.0251
Als1 Δ*als3*_1	Δ*als1* Als3_5	<.0001
Als1 Δ*als3*_1	CAI12_0	<.0001
Als1 Δ*als3*_1	CAI12_1	<.0001
Als1 Δ*als3*_1	CAI12_2	<.0001
Als1 Δ*als3*_1	CAI12_3	0.1077
Als1 Δ*als3*_1	CAI12_4	0.2310
Als1 Δ*als3*_1	CAI12_5	0.0216
Als1 Δ*als3*_2	Als1 Δ*als3*_3	0.0616
Als1 Δ*als3*_2	Als1 Δ*als3*_4	0.2805
Als1 Δ*als3*_2	Als1 Δ*als3*_5	0.0657
Als1 Δ*als3*_2	Δ*als1* Als3_0	0.5474
Als1 Δ*als3*_2	Δ*als1* Als3_1	0.0186
Als1 Δ*als3*_2	Δ*als1* Als3_2	0.0085
Als1 Δ*als3*_2	Δ*als1* Als3_3	<.0001
Als1 Δ*als3*_2	Δ*als1* Als3_4	0.1693
Als1 Δ*als3*_2	Δ*als1* Als3_5	0.4524
Als1 Δ*als3*_2	CAI12_0	0.1603
Als1 Δ*als3*_2	CAI12_1	0.6089
Als1 Δ*als3*_2	CAI12_2	0.0385
Als1 Δ*als3*_2	CAI12_3	0.0441
Als1 Δ*als3*_2	CAI12_4	0.0160
Als1 Δ*als3*_2	CAI12_5	0.1883
Als1 Δ*als3*_3	Als1 Δ*als3*_4	0.0040
Als1 Δ*als3*_3	Als1 Δ*als3*_5	0.0004
Als1 Δ*als3*_3	Δ*als1* Als3_0	0.0148
Als1 Δ*als3*_3	Δ*als1* Als3_1	<.0001
Als1 Δ*als3*_3	Δ*als1* Als3_2	<.0001
Als1 Δ*als3*_3	Δ*als1* Als3_3	<.0001
Als1 Δ*als3*_3	Δ*als1* Als3_4	0.6089
Als1 Δ*als3*_3	Δ*als1* Als3_5	0.0100
Als1 Δ*als3*_3	CAI12_0	0.0015
Als1 Δ*als3*_3	CAI12_1	0.0186
Als1 Δ*als3*_3	CAI12_2	0.0002
Als1 Δ*als3*_3	CAI12_3	0.8803

Als1 Δ*als3*_3	CAI12_4	0.5676
Als1 Δ*als3*_3	CAI12_5	0.5676
Als1 Δ*als3*_4	Als1 Δ*als3*_5	0.4346
Als1 Δ*als3*_4	Δ*als1* Als3_0	0.6301
Als1 Δ*als3*_4	Δ*als1* Als3_1	0.1883
Als1 Δ*als3*_4	Δ*als1* Als3_2	0.1077
Als1 Δ*als3*_4	Δ*als1* Als3_3	<.0001
Als1 Δ*als3*_4	Δ*als1* Als3_4	0.0160
Als1 Δ*als3*_4	Δ*als1* Als3_5	0.7404
Als1 Δ*als3*_4	CAI12_0	0.7404
Als1 Δ*als3*_4	CAI12_1	0.5676
Als1 Δ*als3*_4	CAI12_2	0.3078
Als1 Δ*als3*_4	CAI12_3	0.0026
Als1 Δ*als3*_4	CAI12_4	0.0007
Als1 Δ*als3*_4	CAI12_5	0.0186
Als1 Δ*als3*_5	Δ*als1* Als3_0	0.2088
Als1 Δ*als3*_5	Δ*als1* Als3_1	0.5881
Als1 Δ*als3*_5	Δ*als1* Als3_2	0.4003
Als1 Δ*als3*_5	Δ*als1* Als3_3	<.0001
Als1 Δ*als3*_5	Δ*als1* Als3_4	0.0018
Als1 Δ*als3*_5	Δ*als1* Als3_5	0.2675
Als1 Δ*als3*_5	CAI12_0	0.6516
Als1 Δ*als3*_5	CAI12_1	0.1786
Als1 Δ*als3*_5	CAI12_2	0.8096
Als1 Δ*als3*_5	CAI12_3	0.0002
Als1 Δ*als3*_5	CAI12_4	<.0001
Als1 Δ*als3*_5	CAI12_5	0.0022
Δ*als1* Als3_0	Δ*als1* Als3_1	0.0746
Δ*als1* Als3_0	Δ*als1* Als3_2	0.0385
Δ*als1* Als3_0	Δ*als1* Als3_3	<.0001
Δ*als1* Als3_0	Δ*als1* Als3_4	0.0505
Δ*als1* Als3_0	Δ*als1* Als3_5	0.8803
Δ*als1* Als3_0	CAI12_0	0.4173
Δ*als1* Als3_0	CAI12_1	0.9280
Δ*als1* Als3_0	CAI12_2	0.1357
Δ*als1* Als3_0	CAI12_3	0.0100
Δ*als1* Als3_0	CAI12_4	0.0031
Δ*als1* Als3_0	CAI12_5	0.0577
Δ*als1* Als3_1	Δ*als1* Als3_2	0.7633
Δ*als1* Als3_1	Δ*als1* Als3_3	<.0001
Δ*als1* Als3_1	Δ*als1* Als3_4	0.0003
Δ*als1* Als3_1	Δ*als1* Als3_5	0.1015
Δ*als1* Als3_1	CAI12_0	0.3222

Δ*als1* Als3_1	CAI12_1	0.0616
Δ*als1* Als3_1	CAI12_2	0.7633
Δ*als1* Als3_1	CAI12_3	<.0001
Δ*als1* Als3_1	CAI12_4	<.0001
Δ*als1* Als3_1	CAI12_5	0.0004
Δ*als1* Als3_2	Δ*als1* Als3_3	<.0001
Δ*als1* Als3_2	Δ*als1* Als3_4	0.0001
Δ*als1* Als3_2	Δ*als1* Als3_5	0.0540
Δ*als1* Als3_2	CAI12_0	0.1983
Δ*als1* Als3_2	CAI12_1	0.0311
Δ*als1* Als3_2	CAI12_2	0.5474
Δ*als1* Als3_2	CAI12_3	<.0001
Δ*als1* Als3_2	CAI12_4	<.0001
Δ*als1* Als3_2	CAI12_5	0.0002
Δ*als1* Als3_3	Δ*als1* Als3_4	<.0001
Δ*als1* Als3_3	Δ*als1* Als3_5	<.0001
Δ*als1* Als3_3	CAI12_0	<.0001
Δ*als1* Als3_3	CAI12_1	<.0001
Δ*als1* Als3_3	CAI12_2	<.0001
Δ*als1* Als3_3	CAI12_3	<.0001
Δ*als1* Als3_3	CAI12_4	<.0001
Δ*als1* Als3_3	CAI12_5	<.0001
Δ*als1* Als3_4	Δ*als1* Als3_5	0.0359
Δ*als1* Als3_4	CAI12_0	0.0067
Δ*als1* Als3_4	CAI12_1	0.0616
Δ*als1* Als3_4	CAI12_2	0.0009
Δ*als1* Als3_4	CAI12_3	0.5082
Δ*als1* Als3_4	CAI12_4	0.2805
Δ*als1* Als3_4	CAI12_5	0.9520
Δ*als1* Als3_5	CAI12_0	0.5082
Δ*als1* Als3_5	CAI12_1	0.8096
Δ*als1* Als3_5	CAI12_2	0.1786
Δ*als1* Als3_5	CAI12_3	0.0067
Δ*als1* Als3_5	CAI12_4	0.0020
Δ*als1* Als3_5	CAI12_5	0.0412
CAI12_0	CAI12_1	0.3677
CAI12_0	CAI12_2	0.4892
CAI12_0	CAI12_3	0.0010
CAI12_0	CAI12_4	0.0002
CAI12_0	CAI12_5	0.0079
CAI12_1	CAI12_2	0.1142
CAI12_1	CAI12_3	0.0127
CAI12_1	CAI12_4	0.0040

CAI12_1	CAI12_5	0.0700
CAI12_2	CAI12_3	0.0001
CAI12_2	CAI12_4	<.0001
CAI12_2	CAI12_5	0.0011
CAI12_3	CAI12_4	0.6734
CAI12_3	CAI12_5	0.4706
CAI12_4	CAI12_5	0.2549
