# Supplementary material for: A proposed mechanism for the interaction between the Candida albicans Als3 adhesin and streptococcal cell wall proteins
Source: Front Microbiol. 2014 Nov 4;5:564. doi: 10.3389/fmicb.2014.00564 (PMC4219490; doi:10.3389/fmicb.2014.00564)
Supplement: Supplementary file 3 [file Table3.PDF]

Comparisons Between Means for Fig. 3		
**Observation 1**	**Observation 2**	**P value**
Δ*als3*_0	Δ*als3*_1	0.0004
Δ*als3*_0	Δ*als3*_2	0.0097
Δ*als3*_0	Δ*als3*_3	0.0152
Δ*als3*_0	Δ*als3*_4	<.0001
Δ*als3*_0	Δ*als3*_5	<.0001
Δ*als3*_0	Als3-pbc_0	0.5738
Δ*als3*_0	Als3-pbc_1	<.0001
Δ*als3*_0	Als3-pbc_2	0.0004
Δ*als3*_0	Als3-pbc_3	0.0011
Δ*als3*_0	Als3-pbc_4	<.0001
Δ*als3*_0	Als3-pbc_5	<.0001
Δ*als3*_0	Als3-gk_0	0.0382
Δ*als3*_0	Als3-gk_1	0.0085
Δ*als3*_0	Als3-gk_2	0.0004
Δ*als3*_0	Als3-gk_3	0.9584
Δ*als3*_0	Als3-gk_4	0.0231
Δ*als3*_0	Als3-gk_5	<.0001
Δ*als3*_0	Als3-afr_0	0.0002
Δ*als3*_0	Als3-afr_1	0.8757
Δ*als3*_0	Als3-afr_2	0.0043
Δ*als3*_0	Als3-afr_3	0.7944
Δ*als3*_0	Als3-afr_4	0.4352
Δ*als3*_0	Als3-afr_5	0.0094
Δ*als3*_0	Als3$_{LA}$_0	0.0001
Δ*als3*_0	Als3$_{LA}$_1	0.2178
Δ*als3*_0	Als3$_{LA}$_2	<.0001
Δ*als3*_0	Als3$_{LA}$_3	0.3995
Δ*als3*_0	Als3$_{LA}$_4	0.8218
Δ*als3*_0	Als3$_{LA}$_5	0.0517
Δ*als3*_1	Δ*als3*_2	<.0001
Δ*als3*_1	Δ*als3*_3	<.0001
Δ*als3*_1	Δ*als3*_4	<.0001
Δ*als3*_1	Δ*als3*_5	<.0001
Δ*als3*_1	Als3-pbc_0	0.0023
Δ*als3*_1	Als3-pbc_1	0.0746
Δ*als3*_1	Als3-pbc_2	<.0001
Δ*als3*_1	Als3-pbc_3	<.0001
Δ*als3*_1	Als3-pbc_4	<.0001
Δ*als3*_1	Als3-pbc_5	<.0001
Δ*als3*_1	Als3-gk_0	<.0001
Δ*als3*_1	Als3-gk_1	0.4558

Δ*als3*_1	Als3-gk_2	<.0001
Δ*als3*_1	Als3-gk_3	0.0011
Δ*als3*_1	Als3-gk_4	<.0001
Δ*als3*_1	Als3-gk_5	<.0001
Δ*als3*_1	Als3-afr_0	<.0001
Δ*als3*_1	Als3-afr_1	0.0006
Δ*als3*_1	Als3-afr_2	<.0001
Δ*als3*_1	Als3-afr_3	0.0021
Δ*als3*_1	Als3-afr_4	<.0001
Δ*als3*_1	Als3-afr_5	<.0001
Δ*als3*_1	Als3$_{LA}$_0	<.0001
Δ*als3*_1	Als3$_{LA}$_1	<.0001
Δ*als3*_1	Als3$_{LA}$_2	<.0001
Δ*als3*_1	Als3$_{LA}$_3	0.0052
Δ*als3*_1	Als3$_{LA}$_4	0.0008
Δ*als3*_1	Als3$_{LA}$_5	<.0001
Δ*als3*_2	Δ*als3*_3	0.8659
Δ*als3*_2	Δ*als3*_4	0.1055
Δ*als3*_2	Δ*als3*_5	0.0113
Δ*als3*_2	Als3-pbc_0	0.0019
Δ*als3*_2	Als3-pbc_1	<.0001
Δ*als3*_2	Als3-pbc_2	0.2865
Δ*als3*_2	Als3-pbc_3	0.4650
Δ*als3*_2	Als3-pbc_4	0.0586
Δ*als3*_2	Als3-pbc_5	0.0083
Δ*als3*_2	Als3-gk_0	0.7283
Δ*als3*_2	Als3-gk_1	<.0001
Δ*als3*_2	Als3-gk_2	0.2133
Δ*als3*_2	Als3-gk_3	0.0142
Δ*als3*_2	Als3-gk_4	0.8894
Δ*als3*_2	Als3-gk_5	0.0241
Δ*als3*_2	Als3-afr_0	0.1672
Δ*als3*_2	Als3-afr_1	0.0241
Δ*als3*_2	Als3-afr_2	0.6269
Δ*als3*_2	Als3-afr_3	0.0081
Δ*als3*_2	Als3-afr_4	0.0983
Δ*als3*_2	Als3-afr_5	0.8348
Δ*als3*_2	Als3$_{LA}$_0	0.1792
Δ*als3*_2	Als3$_{LA}$_1	0.1620
Δ*als3*_2	Als3$_{LA}$_2	0.0350
Δ*als3*_2	Als3$_{LA}$_3	0.0008
Δ*als3*_2	Als3$_{LA}$_4	0.0052
Δ*als3*_2	Als3$_{LA}$_5	0.4999

Δ*als3*_3	Δ*als3*_4	0.0746
Δ*als3*_3	Δ*als3*_5	0.0071
Δ*als3*_3	Als3-pbc_0	0.0032
Δ*als3*_3	Als3-pbc_1	<.0001
Δ*als3*_3	Als3-pbc_2	0.2178
Δ*als3*_3	Als3-pbc_3	0.3689
Δ*als3*_3	Als3-pbc_4	0.0400
Δ*als3*_3	Als3-pbc_5	0.0052
Δ*als3*_3	Als3-gk_0	0.8484
Δ*als3*_3	Als3-gk_1	<.0001
Δ*als3*_3	Als3-gk_2	0.1620
Δ*als3*_3	Als3-gk_3	0.0212
Δ*als3*_3	Als3-gk_4	0.9861
Δ*als3*_3	Als3-gk_5	0.0163
Δ*als3*_3	Als3-afr_0	0.1249
Δ*als3*_3	Als3-afr_1	0.0352
Δ*als3*_3	Als3-afr_2	0.5208
Δ*als3*_3	Als3-afr_3	0.0124
Δ*als3*_3	Als3-afr_4	0.1335
Δ*als3*_3	Als3-afr_5	0.7153
Δ*als3*_3	$Als3_{LA}$_0	0.1314
Δ*als3*_3	$Als3_{LA}$_1	0.2178
Δ*als3*_3	$Als3_{LA}$_2	0.0233
Δ*als3*_3	$Als3_{LA}$_3	0.0013
Δ*als3*_3	$Als3_{LA}$_4	0.0083
Δ*als3*_3	$Als3_{LA}$_5	0.6126
Δ*als3*_4	Δ*als3*_5	0.3399
Δ*als3*_4	Als3-pbc_0	<.0001
Δ*als3*_4	Als3-pbc_1	<.0001
Δ*als3*_4	Als3-pbc_2	0.5738
Δ*als3*_4	Als3-pbc_3	0.3689
Δ*als3*_4	Als3-pbc_4	0.7784
Δ*als3*_4	Als3-pbc_5	0.2865
Δ*als3*_4	Als3-gk_0	0.0660
Δ*als3*_4	Als3-gk_1	<.0001
Δ*als3*_4	Als3-gk_2	0.7944
Δ*als3*_4	Als3-gk_3	0.0001
Δ*als3*_4	Als3-gk_4	0.1018
Δ*als3*_4	Als3-gk_5	0.4352
Δ*als3*_4	Als3-afr_0	0.9032
Δ*als3*_4	Als3-afr_1	0.0003
Δ*als3*_4	Als3-afr_2	0.3070
Δ*als3*_4	Als3-afr_3	<.0001

Δ*als3*_4	Als3-afr_4	0.0021
Δ*als3*_4	Als3-afr_5	0.1950
Δ*als3*_4	$Als3_{LA}$_0	0.7784
Δ*als3*_4	$Als3_{LA}$_1	0.0032
Δ*als3*_4	$Als3_{LA}$_2	0.6126
Δ*als3*_4	$Als3_{LA}$_3	<.0001
Δ*als3*_4	$Als3_{LA}$_4	<.0001
Δ*als3*_4	$Als3_{LA}$_5	0.0233
Δ*als3*_5	Als3-pbc_0	<.0001
Δ*als3*_5	Als3-pbc_1	<.0001
Δ*als3*_5	Als3-pbc_2	0.1314
Δ*als3*_5	Als3-pbc_3	0.0662
Δ*als3*_5	Als3-pbc_4	0.4999
Δ*als3*_5	Als3-pbc_5	0.9103
Δ*als3*_5	Als3-gk_0	0.0074
Δ*als3*_5	Als3-gk_1	<.0001
Δ*als3*_5	Als3-gk_2	0.2535
Δ*als3*_5	Als3-gk_3	<.0001
Δ*als3*_5	Als3-gk_4	0.0130
Δ*als3*_5	Als3-gk_5	0.9170
Δ*als3*_5	Als3-afr_0	0.3152
Δ*als3*_5	Als3-afr_1	<.0001
Δ*als3*_5	Als3-afr_2	0.0589
Δ*als3*_5	Als3-afr_3	<.0001
Δ*als3*_5	Als3-afr_4	0.0001
Δ*als3*_5	Als3-afr_5	0.0311
Δ*als3*_5	$Als3_{LA}$_0	0.2178
Δ*als3*_5	$Als3_{LA}$_1	0.0001
Δ*als3*_5	$Als3_{LA}$_2	0.6526
Δ*als3*_5	$Als3_{LA}$_3	<.0001
Δ*als3*_5	$Als3_{LA}$_4	<.0001
Δ*als3*_5	$Als3_{LA}$_5	0.0016
Als3-pbc_0	Als3-pbc_1	<.0001
Als3-pbc_0	Als3-pbc_2	<.0001
Als3-pbc_0	Als3-pbc_3	0.0002
Als3-pbc_0	Als3-pbc_4	<.0001
Als3-pbc_0	Als3-pbc_5	<.0001
Als3-pbc_0	Als3-gk_0	0.0103
Als3-pbc_0	Als3-gk_1	0.0324
Als3-pbc_0	Als3-gk_2	<.0001
Als3-pbc_0	Als3-gk_3	0.6392
Als3-pbc_0	Als3-gk_4	0.0058
Als3-pbc_0	Als3-gk_5	<.0001

Als3-pbc_0	Als3-afr_0	<.0001
Als3-pbc_0	Als3-afr_1	0.4986
Als3-pbc_0	Als3-afr_2	0.0009
Als3-pbc_0	Als3-afr_3	0.7944
Als3-pbc_0	Als3-afr_4	0.1950
Als3-pbc_0	Als3-afr_5	0.0021
Als3-pbc_0	Als3$_{LA}$_0	<.0001
Als3-pbc_0	Als3$_{LA}$_1	0.0746
Als3-pbc_0	Als3$_{LA}$_2	<.0001
Als3-pbc_0	Als3$_{LA}$_3	0.7784
Als3-pbc_0	Als3$_{LA}$_4	0.7356
Als3-pbc_0	Als3$_{LA}$_5	0.0131
Als3-pbc_1	Als3-pbc_2	<.0001
Als3-pbc_1	Als3-pbc_3	<.0001
Als3-pbc_1	Als3-pbc_4	<.0001
Als3-pbc_1	Als3-pbc_5	<.0001
Als3-pbc_1	Als3-gk_0	<.0001
Als3-pbc_1	Als3-gk_1	0.0178
Als3-pbc_1	Als3-gk_2	<.0001
Als3-pbc_1	Als3-gk_3	<.0001
Als3-pbc_1	Als3-gk_4	<.0001
Als3-pbc_1	Als3-gk_5	<.0001
Als3-pbc_1	Als3-afr_0	<.0001
Als3-pbc_1	Als3-afr_1	<.0001
Als3-pbc_1	Als3-afr_2	<.0001
Als3-pbc_1	Als3-afr_3	<.0001
Als3-pbc_1	Als3-afr_4	<.0001
Als3-pbc_1	Als3-afr_5	<.0001
Als3-pbc_1	Als3$_{LA}$_0	<.0001
Als3-pbc_1	Als3$_{LA}$_1	<.0001
Als3-pbc_1	Als3$_{LA}$_2	<.0001
Als3-pbc_1	Als3$_{LA}$_3	<.0001
Als3-pbc_1	Als3$_{LA}$_4	<.0001
Als3-pbc_1	Als3$_{LA}$_5	<.0001
Als3-pbc_2	Als3-pbc_3	0.7356
Als3-pbc_2	Als3-pbc_4	0.3995
Als3-pbc_2	Als3-pbc_5	0.1055
Als3-pbc_2	Als3-gk_0	0.1835
Als3-pbc_2	Als3-gk_1	<.0001
Als3-pbc_2	Als3-gk_2	0.7944
Als3-pbc_2	Als3-gk_3	0.0008
Als3-pbc_2	Als3-gk_4	0.2607
Als3-pbc_2	Als3-gk_5	0.1950

Als3-pbc_2	Als3-afr_0	0.6896
Als3-pbc_2	Als3-afr_1	0.0015
Als3-pbc_2	Als3-afr_2	0.6146
Als3-pbc_2	Als3-afr_3	0.0004
Als3-pbc_2	Als3-afr_4	0.0094
Als3-pbc_2	Als3-afr_5	0.4352
Als3-pbc_2	$Als3_{LA}$_0	0.7784
Als3-pbc_2	$Als3_{LA}$_1	0.0152
Als3-pbc_2	$Als3_{LA}$_2	0.2865
Als3-pbc_2	$Als3_{LA}$_3	<.0001
Als3-pbc_2	$Als3_{LA}$_4	0.0002
Als3-pbc_2	$Als3_{LA}$_5	0.0840
Als3-pbc_3	Als3-pbc_4	0.2392
Als3-pbc_3	Als3-pbc_5	0.0517
Als3-pbc_3	Als3-gk_0	0.3070
Als3-pbc_3	Als3-gk_1	<.0001
Als3-pbc_3	Als3-gk_2	0.5668
Als3-pbc_3	Als3-gk_3	0.0021
Als3-pbc_3	Als3-gk_4	0.4151
Als3-pbc_3	Als3-gk_5	0.1091
Als3-pbc_3	Als3-afr_0	0.4769
Als3-pbc_3	Als3-afr_1	0.0039
Als3-pbc_3	Als3-afr_2	0.8484
Als3-pbc_3	Als3-afr_3	0.0011
Als3-pbc_3	Als3-afr_4	0.0212
Als3-pbc_3	Als3-afr_5	0.6392
Als3-pbc_3	$Als3_{LA}$_0	0.5362
Als3-pbc_3	$Als3_{LA}$_1	0.0350
Als3-pbc_3	$Als3_{LA}$_2	0.1620
Als3-pbc_3	$Als3_{LA}$_3	<.0001
Als3-pbc_3	$Als3_{LA}$_4	0.0005
Als3-pbc_3	$Als3_{LA}$_5	0.1620
Als3-pbc_4	Als3-pbc_5	0.4315
Als3-pbc_4	Als3-gk_0	0.0367
Als3-pbc_4	Als3-gk_1	<.0001
Als3-pbc_4	Als3-gk_2	0.6025
Als3-pbc_4	Als3-gk_3	<.0001
Als3-pbc_4	Als3-gk_4	0.0589
Als3-pbc_4	Als3-gk_5	0.6025
Als3-pbc_4	Als3-afr_0	0.7024
Als3-pbc_4	Als3-afr_1	0.0001
Als3-pbc_4	Als3-afr_2	0.2010
Als3-pbc_4	Als3-afr_3	<.0001

Als3-pbc_4	Als3-afr_4	0.0009
Als3-pbc_4	Als3-afr_5	0.1208
Als3-pbc_4	$Als3_{LA}$_0	0.5738
Als3-pbc_4	$Als3_{LA}$_1	0.0013
Als3-pbc_4	$Als3_{LA}$_2	0.8218
Als3-pbc_4	$Als3_{LA}$_3	<.0001
Als3-pbc_4	$Als3_{LA}$_4	<.0001
Als3-pbc_4	$Als3_{LA}$_5	0.0113
Als3-pbc_5	Als3-gk_0	0.0055
Als3-pbc_5	Als3-gk_1	<.0001
Als3-pbc_5	Als3-gk_2	0.2133
Als3-pbc_5	Als3-gk_3	<.0001
Als3-pbc_5	Als3-gk_4	0.0098
Als3-pbc_5	Als3-gk_5	0.8348
Als3-pbc_5	Als3-afr_0	0.2681
Als3-pbc_5	Als3-afr_1	<.0001
Als3-pbc_5	Als3-afr_2	0.0467
Als3-pbc_5	Als3-afr_3	<.0001
Als3-pbc_5	Als3-afr_4	<.0001
Als3-pbc_5	Als3-afr_5	0.0241
Als3-pbc_5	$Als3_{LA}$_0	0.1792
Als3-pbc_5	$Als3_{LA}$_1	<.0001
Als3-pbc_5	$Als3_{LA}$_2	0.5738
Als3-pbc_5	$Als3_{LA}$_3	<.0001
Als3-pbc_5	$Als3_{LA}$_4	<.0001
Als3-pbc_5	$Als3_{LA}$_5	0.0011
Als3-gk_0	Als3-gk_1	<.0001
Als3-gk_0	Als3-gk_2	0.1377
Als3-gk_0	Als3-gk_3	0.0467
Als3-gk_0	Als3-gk_4	0.8454
Als3-gk_0	Als3-gk_5	0.0154
Als3-gk_0	Als3-afr_0	0.1071
Als3-gk_0	Als3-afr_1	0.0717
Als3-gk_0	Als3-afr_2	0.4362
Als3-gk_0	Als3-afr_3	0.0296
Als3-gk_0	Als3-afr_4	0.2190
Als3-gk_0	Als3-afr_5	0.6033
Als3-gk_0	$Als3_{LA}$_0	0.1129
Als3-gk_0	$Als3_{LA}$_1	0.3407
Als3-gk_0	$Als3_{LA}$_2	0.0222
Als3-gk_0	$Als3_{LA}$_3	0.0050
Als3-gk_0	$Als3_{LA}$_4	0.0231
Als3-gk_0	$Als3_{LA}$_5	0.7810

Als3-gk_1	Als3-gk_2	<.0001
Als3-gk_1	Als3-gk_3	0.0154
Als3-gk_1	Als3-gk_4	<.0001
Als3-gk_1	Als3-gk_5	<.0001
Als3-gk_1	Als3-afr_0	<.0001
Als3-gk_1	Als3-afr_1	0.0092
Als3-gk_1	Als3-afr_2	<.0001
Als3-gk_1	Als3-afr_3	0.0253
Als3-gk_1	Als3-afr_4	0.0017
Als3-gk_1	Als3-afr_5	<.0001
Als3-gk_1	$Als3_{LA}$_0	<.0001
Als3-gk_1	$Als3_{LA}$_1	0.0002
Als3-gk_1	$Als3_{LA}$_2	<.0001
Als3-gk_1	$Als3_{LA}$_3	0.0589
Als3-gk_1	$Als3_{LA}$_4	0.0149
Als3-gk_1	$Als3_{LA}$_5	<.0001
Als3-gk_2	Als3-gk_3	0.0007
Als3-gk_2	Als3-gk_4	0.1960
Als3-gk_2	Als3-gk_5	0.3310
Als3-gk_2	Als3-afr_0	0.8965
Als3-gk_2	Als3-afr_1	0.0014
Als3-gk_2	Als3-afr_2	0.4752
Als3-gk_2	Als3-afr_3	0.0004
Als3-gk_2	Als3-afr_4	0.0077
Als3-gk_2	Als3-afr_5	0.3310
Als3-gk_2	$Als3_{LA}$_0	1.0000
Als3-gk_2	$Als3_{LA}$_1	0.0124
Als3-gk_2	$Als3_{LA}$_2	0.4663
Als3-gk_2	$Als3_{LA}$_3	<.0001
Als3-gk_2	$Als3_{LA}$_4	0.0002
Als3-gk_2	$Als3_{LA}$_5	0.0636
Als3-gk_3	Als3-gk_4	0.0296
Als3-gk_3	Als3-gk_5	<.0001
Als3-gk_3	Als3-afr_0	0.0005
Als3-gk_3	Als3-afr_1	0.8454
Als3-gk_3	Als3-afr_2	0.0064
Als3-gk_3	Als3-afr_3	0.8454
Als3-gk_3	Als3-afr_4	0.4362
Als3-gk_3	Als3-afr_5	0.0130
Als3-gk_3	$Als3_{LA}$_0	0.0003
Als3-gk_3	$Als3_{LA}$_1	0.2328
Als3-gk_3	$Als3_{LA}$_2	<.0001
Als3-gk_3	$Als3_{LA}$_3	0.4663

Als3-gk_3	$Als3_{LA}$_4	0.8757
Als3-gk_3	$Als3_{LA}$_5	0.0636
Als3-gk_4	Als3-gk_5	0.0253
Als3-gk_4	Als3-afr_0	0.1554
Als3-gk_4	Als3-afr_1	0.0467
Als3-gk_4	Als3-afr_2	0.5589
Als3-gk_4	Als3-afr_3	0.0182
Als3-gk_4	Als3-afr_4	0.1554
Als3-gk_4	Als3-afr_5	0.7452
Als3-gk_4	$Als3_{LA}$_0	0.1672
Als3-gk_4	$Als3_{LA}$_1	0.2465
Als3-gk_4	$Als3_{LA}$_2	0.0367
Als3-gk_4	$Als3_{LA}$_3	0.0027
Als3-gk_4	$Als3_{LA}$_4	0.0136
Als3-gk_4	$Als3_{LA}$_5	0.6269
Als3-gk_5	Als3-afr_0	0.3991
Als3-gk_5	Als3-afr_1	<.0001
Als3-gk_5	Als3-afr_2	0.0940
Als3-gk_5	Als3-afr_3	<.0001
Als3-gk_5	Als3-afr_4	0.0004
Als3-gk_5	Als3-afr_5	0.0541
Als3-gk_5	$Als3_{LA}$_0	0.2989
Als3-gk_5	$Als3_{LA}$_1	0.0006
Als3-gk_5	$Als3_{LA}$_2	0.7545
Als3-gk_5	$Als3_{LA}$_3	<.0001
Als3-gk_5	$Als3_{LA}$_4	<.0001
Als3-gk_5	$Als3_{LA}$_5	0.0045
Als3-afr_0	Als3-afr_1	0.0009
Als3-afr_0	Als3-afr_2	0.3991
Als3-afr_0	Als3-afr_3	0.0002
Als3-afr_0	Als3-afr_4	0.0053
Als3-afr_0	Als3-afr_5	0.2710
Als3-afr_0	$Als3_{LA}$_0	0.8894
Als3-afr_0	$Als3_{LA}$_1	0.0085
Als3-afr_0	$Als3_{LA}$_2	0.5551
Als3-afr_0	$Als3_{LA}$_3	<.0001
Als3-afr_0	$Als3_{LA}$_4	0.0001
Als3-afr_0	$Als3_{LA}$_5	0.0467
Als3-afr_1	Als3-afr_2	0.0109
Als3-afr_1	Als3-afr_3	0.6966
Als3-afr_1	Als3-afr_4	0.5589
Als3-afr_1	Als3-afr_5	0.0215
Als3-afr_1	$Als3_{LA}$_0	0.0007

Als3-afr_1	$Als3_{LA}$_1	0.3235
Als3-afr_1	$Als3_{LA}$_2	<.0001
Als3-afr_1	$Als3_{LA}$_3	0.3495
Als3-afr_1	$Als3_{LA}$_4	0.7153
Als3-afr_1	$Als3_{LA}$_5	0.0983
Als3-afr_2	Als3-afr_3	0.0037
Als3-afr_2	Als3-afr_4	0.0467
Als3-afr_2	Als3-afr_5	0.7949
Als3-afr_2	$Als3_{LA}$_0	0.4454
Als3-afr_2	$Als3_{LA}$_1	0.0766
Als3-afr_2	$Als3_{LA}$_2	0.1380
Als3-afr_2	$Als3_{LA}$_3	0.0004
Als3-afr_2	$Als3_{LA}$_4	0.0023
Als3-afr_2	$Als3_{LA}$_5	0.2681
Als3-afr_3	Als3-afr_4	0.3310
Als3-afr_3	Als3-afr_5	0.0077
Als3-afr_3	$Als3_{LA}$_0	0.0002
Als3-afr_3	$Als3_{LA}$_1	0.1620
Als3-afr_3	$Als3_{LA}$_2	<.0001
Als3-afr_3	$Als3_{LA}$_3	0.6025
Als3-afr_3	$Als3_{LA}$_4	0.9584
Als3-afr_3	$Als3_{LA}$_5	0.0398
Als3-afr_4	Als3-afr_5	0.0823
Als3-afr_4	$Als3_{LA}$_0	0.0045
Als3-afr_4	$Als3_{LA}$_1	0.7153
Als3-afr_4	$Als3_{LA}$_2	0.0005
Als3-afr_4	$Als3_{LA}$_3	0.1208
Als3-afr_4	$Als3_{LA}$_4	0.3235
Als3-afr_4	$Als3_{LA}$_5	0.2989
Als3-afr_5	$Als3_{LA}$_0	0.2989
Als3-afr_5	$Als3_{LA}$_1	0.1335
Als3-afr_5	$Als3_{LA}$_2	0.0794
Als3-afr_5	$Als3_{LA}$_3	0.0009
Als3-afr_5	$Als3_{LA}$_4	0.0052
Als3-afr_5	$Als3_{LA}$_5	0.4053
$Als3_{LA}$_0	$Als3_{LA}$_1	0.0071
$Als3_{LA}$_0	$Als3_{LA}$_2	0.4315
$Als3_{LA}$_0	$Als3_{LA}$_3	<.0001
$Als3_{LA}$_0	$Als3_{LA}$_4	<.0001
$Als3_{LA}$_0	$Als3_{LA}$_5	0.0455
$Als3_{LA}$_1	$Als3_{LA}$_2	0.0006
$Als3_{LA}$_1	$Als3_{LA}$_3	0.0400
$Als3_{LA}$_1	$Als3_{LA}$_4	0.1461

Als3$_{LA}$_1	Als3$_{LA}$_5	0.4650
Als3$_{LA}$_2	Als3$_{LA}$_3	<.0001
Als3$_{LA}$_2	Als3$_{LA}$_4	<.0001
Als3$_{LA}$_2	Als3$_{LA}$_5	0.0061
Als3$_{LA}$_3	Als3$_{LA}$_4	0.5362
Als3$_{LA}$_3	Als3$_{LA}$_5	0.0061
Als3$_{LA}$_4	Als3$_{LA}$_5	0.0306
